# Self-assembly of differentiated progenitor cells facilitates spheroid human skin organoid formation and planar skin regeneration

**DOI:** 10.7150/thno.59661

**Published:** 2021-07-25

**Authors:** Patricia Ebner-Peking, Linda Krisch, Martin Wolf, Sarah Hochmann, Anna Hoog, Balázs Vári, Katharina Muigg, Rodolphe Poupardin, Cornelia Scharler, Sabine Schmidhuber, Elisabeth Russe, Harald Stachelscheid, Achim Schneeberger, Katharina Schallmoser, Dirk Strunk

**Affiliations:** 1 Cell Therapy Institute, Spinal Cord Injury and Tissue Regeneration Center Salzburg (SCI-TReCS), University Clinic, Paracelsus Medical University, Salzburg, Austria; 2 Department of Transfusion Medicine, University Clinic, Paracelsus Medical University, Salzburg, Austria; 3 Accanis Biotech, Biocenter Vienna, Austria; 4 Department of Plastic, Aesthetic and Reconstructive Surgery, Hospital Barmherzige Brueder, Salzburg, Austria; 5 Stem Cell Core Facility, Charité, Berlin Institute of Health; Berlin, Germany

**Keywords:** Skin organoids, progenitor cells, human induced pluripotent stem cells, human platelet-derived growth factors, skin regeneration

## Abstract

Self-assembly of solid organs from single cells would greatly expand applicability of regenerative medicine. Stem/progenitor cells can self-organize into micro-sized organ units, termed organoids, partially modelling tissue function and regeneration. Here we demonstrated 3D self-assembly of adult and induced pluripotent stem cell (iPSC)-derived fibroblasts, keratinocytes and endothelial progenitors into both, planar human skin in vivo and a novel type of spheroid-shaped skin organoids in vitro, under the aegis of human platelet lysate.

**Methods:** Primary endothelial colony forming cells (ECFCs), skin fibroblasts (FBs) and keratinocytes (KCs) were isolated from human tissues and polyclonally propagated under 2D xeno-free conditions. Human tissue-derived iPSCs were differentiated into endothelial cells (hiPSC-ECs), fibroblasts (hiPSC-FBs) and keratinocytes (hiPSC-KCs) according to efficiency-optimized protocols. Cell identity and purity were confirmed by flow cytometry and clonogenicity indicated their stem/progenitor potential. Triple cell type floating spheroids formation was promoted by human platelet-derived growth factors containing culture conditions, using nanoparticle cell labelling for monitoring the organization process. Planar human skin regeneration was assessed in full-thickness wounds of immune-deficient mice upon transplantation of hiPSC-derived single cell suspensions.

**Results:** Organoids displayed a distinct architecture with surface-anchored keratinocytes surrounding a stromal core, and specific signaling patterns in response to inflammatory stimuli. FGF-7 mRNA transfection was required to accelerate keratinocyte long-term fitness. Stratified human skin also self-assembled within two weeks after either adult- or iPSC-derived skin cell-suspension liquid-transplantation, healing deep wounds of mice. Transplant vascularization significantly accelerated in the presence of co-transplanted endothelial progenitors. Mechanistically, extracellular vesicles mediated the multifactorial platelet-derived trophic effects. No tumorigenesis occurred upon xenografting.

**Conclusion:** This illustrates the superordinate progenitor self-organization principle and permits novel rapid 3D skin-related pharmaceutical high-content testing opportunities with floating spheroid skin organoids. Multi-cell transplant self-organization facilitates development of iPSC-based organ regeneration strategies using cell suspension transplantation supported by human platelet factors.

## Introduction

Stem/progenitor cell-derived 3D organoids have become standard tools for biomedical research and regenerative medicine 1,2. Appropriate vascularization was recently recognized to better mimic complex organ architecture in organoid test systems and perhaps even create instantly transplantable tissue 3-7. Conventional planar skin equivalents, though 3D, are not necessarily considered to represent a predecessor of current spheroid organoids. Skin equivalents were described in the 1980s based on pioneering co-cultures of keratinocytes on fibroblasts at the air-liquid interface, basically established by Rheinwald and Green 8. Refined skin equivalents are meanwhile validated and licensed tools, upon elaborate 5-6-week manufacturing, for basic research and toxicity testing, replacing animal experimentation as part of global 3R efforts 9,10. Focusing on hair regeneration as a demanding task, three landmark studies have taken different paths. Self-organization of mouse neonatal skin cells into planar hair-bearing skin via an early organoid-like aggregation state was demonstrated to be orchestrated by sequential action of growth factors and Wnt signalling 11. Mouse hair follicle development was established as a model for skin development creating de novo hair follicles from mouse embryonic stem cells in a process that mimics embryonic hair folliculogenesis 12. Most recently, complex organoids containing hairy skin with appendages, recapitulating second trimester human tissue, were generated from human iPSCs (hiPSCs) with a sophisticated protocol over 4-5-month incubation periods by the same team. After removing contaminating cartilage and other extracutaneous structures, approximately half of these tissues even established hairy human skin on mice, opening entirely new opportunities to study human skin development 13.

The human skin is the body's largest organ, fulfilling multiple biochemical and sensory functions in addition to forming the physical body boundary. Locally damaged adult skin can heal with scars but it lacks the capacity to fully regenerate the complex composition of larger skin areas 14. Severe scarring and chronic wounds after major skin burns 15, surgeries or devastating skin diseases 16 limit skin mobility, respiration and light protection, resulting in body fluid loss, life-threatening infections and skin cancer 17,18. The current gold standard for extended area epidermal replacement is transplantation of ex vivo engineered epidermal sheets 19,20. Improved dermal restoration and vascularization strategies are prerequisite for providing an oxygen-rich environment that promotes extended skin tissue regeneration 21,22**.** In vitro generated dermis equivalents with pre-formed vessels showed enhanced full-thickness skin wound repair in rats 23,24. Transplantation of endothelial cells could induce neo-vascularization upon co-transplantation of stromal cells that acted as pericytes stabilizing de-novo formed vessels and reducing endothelial immunogenicity 25-28. Platelet-derived regenerative factors promoted angiogenesis, collagen synthesis and epithelialization 29,30. Adult cells as a source of multicell-transplants offer ease of extraction and lack pluripotency-related teratoma risk but are limited in number. The use of hiPSCs for tissue regeneration has great potential due to their proliferative and differentiation capacity generating virtually unlimited cell amounts including somatic as well as stromal and endothelial cells 31. Various cell types can be generated from a single hiPSC clone, making the reconstruction of complex organs a realistic vision.

Extended time to create transplantable skin equivalents, missing pre-vascularization and lack of superior outcome in clinical testing compared to invasively obtained split skin transplants have hampered broader clinical applicability so far 32. Self-organization of cells into three-dimensional multicellular organoid-like structures may be enhanced by advanced bioengineering strategies including 3D printing 33. Towards scar-free skin regeneration, various improvements still need to be achieved to get regionalized skin comprizing loco-typic hairiness and appendages. Ideally this will enable creation of perfect organoid models to read-out complex transcriptional regulatory networks in their response to environmental or pharmacologic input signals 34,35. It remains to be clarified, to what extent the rather simplistic avascular organoids can facilitate intercellular communication analysis and development of novel pharmacologic or diagnostic strategies 36.

Here we introduce a novel strategy essentially based on rapid skin progenitor self-assembly into (1) vascularized spheroid floating skin organoids (FSOs) in vitro and (2) properly stratified rapidly vascularized human skin in vivo. The complex mixture of platelet-derived growth factors in human platelet lysate (hPL) acted in a dose-dependent manner to orchestrate self-organization and de novo transplant vasculogenesis, virtually mimicking wound repair processes 30 in vitro and, after co-transplantation of human endothelial cells with fibroblasts and keratinocytes, in vivo. Comparative molecular response profiling showed distinct signaling patterns in organoids that differed from separated cell types. Both, human adult progenitor cell types and hiPSC-derived matured skin cells self-organized within transplanted cell suspensions inside a transplant chamber healing deep skin wounds.

## Material and Methods

### Ethics statement and animal welfare

Permissions for human blood and marrow cell collection as well as genetic reprogramming were obtained from the Institutional Review Board of the Medical University of Graz (protocols EK 19-252, EK 21-060) and the Ethics Committee of the province of Salzburg (protocol 415-E/1776/4-2014). Samples were collected after written informed consent from healthy volunteers according to the Declaration of Helsinki. Human full-thickness skin was obtained as biological waste material after informed consent as approved by the ethical committee of the region of Salzburg (vote number: 415-E/1990/8-216). Animal trial permission was granted according to Austrian legislation (§26 TVG 2012; animal trial number: BMBWF-66.019/0032-V/3b/2018). Neonatal human dermal fibroblasts were used from different donors (sample IDs 0000480241, 0000480240; CC-2509, Lonza). The adult foreskin fibroblast-derived hiPSC line BIHi001-A was registered and described in detail at https://hpscreg.eu/cell-line/BIHi001-A. Adult bone marrow- and neonatal umbilical cord blood-derived hiPSCs were described elsewhere 37. All hiPSC strains in this study were created by using non-integrating Sendai virus-based reprogramming.

### Isolation and 2D propagation of primary cells

Keratinocytes and fibroblasts were isolated from human full-thickness skin from male and female healthy donors with ages ranging from 23 to 56 years (8 donors total). Split-thickness human skin with an area of 5 x 5 cm² was generated using an Acculan 3 Ti-Dermatome (GA670, B. Braun), and incubated in dispase (4942078001, Roche) to separate epidermis from dermis. Epidermal sheets were digested with TrypLE (12605028, Gibco) to release the keratinocytes for subsequent culture in collagen I-coated (C3867, Sigma) plates in epithelial culture medium CntPrime (CnT-PR, CELLnTEC). For in vitro and in vivo experiments, exclusively high proliferative keratinocytes were used. Fibroblasts grew out from the dermis upon incubating punch biopsies in α-MEM (4526, Sigma) supplemented with 10% pooled hPL, 0.6% N(2)-L-alanyl-L-glutamin (Dipeptiven^®^, Fresenius Kabi), 0.04% heparin (BC-L6510, Biochrom) and 1% penicillin-streptomycin (P0781, Sigma) (α-MEM/10% hPL) as described 38. ECFCs were isolated from umbilical cord blood (3 donors) according to an established protocol 39 and propagated in EGM basal medium (CC-3156, Lonza) including all supplements as provided by the manufacturer (EGM-2 medium, CC4176, Lonza) and replacing FBS with hPL. All cells were cultured in humidified nitrogen-controlled incubators at 37°C, 5% O_2_ and 5% CO_2_, and were large-scale expanded in four-layered cell factories (CF-4, 140360, Nunc/Thermo). For selected animal experiments, fibroblasts were cultured for comparison in α-MEM/10% FBS, otherwise supplemented as described above, whenever specified in the results section.

### Human iPSCs generation and differentiation

Human iPSCs were reprogrammed from primary stromal cells derived from bone marrow and umbilical cord blood using a non-integrative Sendai viral vector kit (A1378001, Life Technologies) at the Harvard Stem Cell Institute (HSCI) iPSC Core Facility (Cambridge, MA, USA) adapted from an established protocol 40 and characterized in a previous project 37. In addition, we used hiPSCs from dermal fibroblasts (BIHi001-A), generated and characterized by the Stem Cell Core Facility, Charité, Berlin Institute of Health. Human iPSCs were cultured on Matrigel^®^ (354234, Corning) in mTeSR^TM^1 (85850, STEMCELL Technologies) medium. Large-scale expansion to generate a working cell bank for subsequent experiments was performed in four-layered cell factories. For ectoderm induction, 30% confluent dermal and bone marrow derived hiPSC colonies were propagated in keratinocyte serum-free medium (17005042, ThermoFisher) supplemented with 1 µM retinoic acid (R2625, Sigma) and 25 ng/ml bone morphogenetic protein-4 (SRP3016, Sigma) for 6 days performing a media change every second day according to published work 41. In addition, the cells were transfected with 1 µg keratinocyte growth factor (KGF) stabilized mRNA (TriLink) at day 0 and day 2 during differentiation. Keratinocyte maturation and expansion were performed in Cnt-Prime epithelial culture medium for approximately 30 days. Mesoderm induction and fibroblast maturation from bone marrow and umbilical cord blood derived hiPSCs, respectively, were performed based on an established protocol 37. The maturation phase was extended from passage 4 to passage >8 in α-MEM/10% hPL to obtain mature CD90^+^ hiPSC-FBs. Bone marrow and umbilical cord blood derived hiPSCs were used for endothelial differentiation according to an established protocol with modifications 42. In brief, 5 x 10^4^ hiPSCs per cm² were seeded as single cells followed by mesoderm induction using the mesoderm induction medium (05221, STEMCELL Technologies) for 48 hours. Thereafter, the cells were exposed to EGM-2/10% hPL with 260 ng/ml vascular endothelial growth factor (293-VE, R&D Systems) and 2 µM forskolin (F6886, Sigma) for 5 days prior to cell sorting. After sorting, cells were cultured in EGM-2/10% hPL and expanded for characterization, functional assays and transplantation.

### Transfection of in vitro transcribed stabilized FGF-7 mRNA

In order to enhance ectoderm induction and improve long-term growth of hiPSC-KCs, the hiPSCs were transfected simultaneously with eGFP (as a reporter) and stabilized FGF-7 mRNA (TriLink BioTechnologies). The FGF-7 sequence was obtained from https://www.ensembl.org/index.html and produced by using wild type bases. Modification to stabilize the mRNA was done by capping (Cap 1) using CleanCap^TM^ AG / Polyadenylation (120A). Upon DNAse and phosphatase treatment, the mRNA was purified using a silica membrane. Transfection of hiPSC colonies was done using Lipofectamine MessengerMAX transfection reagent (LMRNA003, ThermoFisher) as described 38.

FGF-7 protein expression was measured in a time course after FGF-7 mRNA transfection as specified in the results section. For each time point, supernatant was collected and cell protein lysate was isolated using a RIPA Lysis buffer (R0278, Sigma) including a protein inhibitor (87786, Life Technologies). ELISA measurement was performed according to the manufacturer's protocol (DuoSet ELISA, DY251, R&D Systems) using an OD of 450 nm. The optical density values of undiluted and diluted (1:10) samples were calculated using a linear standard curve. Cell proliferation was evaluated by using the Click-iT^TM^ EdU Cell Proliferation Kit; Alexa Fluor^TM^ 594 dye (C10339, ThermoFisher) according to the recommended protocol. Cells were EdU labelled at passage one and two during the maturation phase for 16 hours, harvested and analyzed by flow cytometry (see section below) and fluorescence microscopy.

### Flow cytometry and cell sorting

The phenotype of primary cells, hiPSCs and hiPSC-derived cells was identified by flow cytometry using various surface and intracellular antibodies (**see Table S4**). Flow cytometry was performed as previously described 26,43,44 with a five-laser BD LSR-Fortessa^TM^ (BD Biosciences), BD FACSDiva Software 8.0.1 Firmware version 1.4 and Kaluza analysis software version 1.3.14026.13330 (Beckman Coulter). In order to obtain pure CD31^+^ endothelial cell populations after seven days of differentiation, cells were labelled with anti-human CD31-eFluor 450 (clone WM59, eBioscience) and subjected to sterile fluorescence-activated cell sorting, using a BD FACSAria^TM^ III instrument (BD Biosciences) and BD FACSDiva Software 8.0.1 Firmware version 1.3.

### Immunofluorescence staining of differentiated cells in situ

For cell characterization, 1 x 10^5^ primary hiPSCs or hiPSC-derived cells were seeded in 12-well plates, grown to 50 - 70% confluence and fixed with 4% formaldehyde for 10 minutes at room temperature. Cells were washed in PBS and permeabilized in citrate buffer and blocked with 1x Dako wash buffer/10% FBS (S300685-2, Agilent). Anti-human CD90-PE (40 ng/µl, clone 5E10, BD Biosciences), anti-human CD31-PE (12.5 ng/µl, clone WM59, BD Biosciences), anti-human Cytokeratin 14 (4 ng/µl, clone LL001, Santa Cruz), anti-human Cytokeratin 5 (20 ng/µl, clone 2C2, Thermo Fischer) primary antibodies and appropriately titrated isotype controls were applied overnight at 4°C. As secondary antibody, a goat anti-mouse PE (40 ng/µl, BD Biosciences) was applied for 1 hour at room temperature. Cell nuclei were stained with 4′,6-Diamidin-2-phenylindol (DAPI, 1:1000, D1306, Molecular Probes) at room temperature for 10 minutes. In situ reporter staining during endothelial cell differentiation was performed by adding 125 ng/ml of anti-human CD31-PE (clone WM59, BD Biosciences) antibody in basal medium and incubating for 30 min at 37°C. After washing the cells with basal medium, EGM-2 / 10% hPL was added back before reporter staining was analyzed by fluorescence imaging.

### Spheroid and floating spheroid skin organoid (FSO) formation

Fibroblasts, keratinocytes and endothelial cells were labelled with cell tracker fluorescent probes (C2110 excitation (ex) / emission (em) 353/466 nm, C7025 ex/em 492/517 nm, C34552 ex/em 577/602 nm; ThermoFisher) according to the producer's recommended protocol. The spheroid formation capacity of each cell type separately was evaluated by seeding 4 x 10^5^ cells in 6-well ultra-low attachment culture plates (CLS3471; Corning) in the respective propagation media. For FSO formation, single-cell suspension consisting of 1.25 x 10^5^ fibroblasts, 1.25 x 10^5^ endothelial cells and 2.5 x 10^5^ keratinocytes were prepared based on preliminary titration and seeded in various media conditions: (i) keratinocyte serum-free medium basal (17005075, Gibco), (ii) keratinocyte serum-free medium basal + supplements (EGF + BPE), (iii) keratinocyte serum-free medium basal + supplements + 10% hPL, (iv) endothelial cell basal medium (EBM, CC-3156, Lonza), (v) endothelial growth medium (EGM) = EBM + supplements (EGF, IGF, hFGF, VEGF, hydrocortisone, ascorbic acid; CC4176, Lonza), (vi) EGM incl. supplements + 10% hPL. Five days after cell seeding, areas of 1 mm² were counted to determine organoid numbers. Life cell imaging was performed for the first 48 hours; microscopic analysis was done 5 days after seeding (see image acquisition).

### Proteome profiler arrays

Protein isolation and proteome profiler arrays (Human Phospho Kinase array Kit, ARY003C, Human Phospho-RTK Kit, ARY001B; Proteome Profiler Human NFkB Pathway Array, ARY029; all R&D Systems) were performed according to the manufacturer's protocol comparing stimulation vs. solvent treatment. In brief, for the human Phospho Kinase array, FSOs, fibroblasts, keratinocytes and endothelial cells were starved in serum-free medium for 2 hours before stimulation with 100 ng/ml recombinant human Interleukin 17A (317-ILB-050, R&D Systems) dissolved in 4 mM HCL. For the human Phospho-RTK Kit and the human NFkB Pathway Array non-starved cells and organoids were used. Protein content was measured using a DC Protein Assay Kit II (500-0112, BioRad) according to the manufacturers protocol. Membrane dots were visualized and quantified using a Chemidoc system and image lab 6.0.1 software (all Bio-Rad).

### Single-cell suspension grafting

For in vivo grafting, immune-deficient NOD.Cg-Prkdcscid Il2rgtm1WjI/SzJ mice (614 NSG, Charles River) were used at an age of 9, 12, 17, 19, 20, 21 and 24 weeks. Full-thickness skin wounds covering app. 2.2% of the body surface area were induced by 8 mm punch biopsy (48801, PFM medical) in the back skin. A silicone grafting chamber including three pores of 2 mm diameter for transplantation and subsequent air contact (50.2 mm² silicone chamber, self-produced; see **Figure 3A**) was placed on the muscle fascia into the wound. For initial experiments, single-cell suspension transplants consisting of 6 x 10^6^ keratinocytes and 6 x 10^6^ fibroblasts diluted in 200 µl of α-MEM/10% FBS (total volume 400 µl) were transplanted as adapted from a published protocol 45. In order to enhance the grafting strategy, FBS was compared to 1%, 10% or 100% hPL and 3 x 10^6^ endothelial cells were co-transplanted as indicated together with 3 x 10^6^ fibroblasts and 6 x 10^6^ keratinocytes. In addition, hiPSC-derived single-cell suspensions consisting of 6 x 10^6^ hiPSC-KCs, 3 x 10^6^ hiPSC-FBs and 3 x 10^6^ hiPSC-ECs, diluted in 200 µl of α-MEM/10% hPL, were grafted. The chambers were removed seven days after transplantation. Skin biopsies were taken at 14 days, 28 days and 3 months after initial grafting, fixed in 4% formaldehyde and prepared for histology.

### Histology of skin sections and FSOs

Paraffin-embedded skin and organoid samples were cut into 4 µm sections for immunofluorescence, histochemistry and immune-histochemistry. For evaluating the human origin of skin cell grafts, a mouse monoclonal anti-human vimentin antibody (1.56 µg/mL, clone V9, M072501-2, Dako), a rabbit monoclonal anti-human CD44 (0.7 µg/mL, SP37, Ventana) and a mouse monoclonal anti-EGFR (1 µg/mL, 3C6, Ventana) were used. Murine erythrocytes were identified using a rat monoclonal anti-murine Ter119-PE antibody (40 ng/µl, clone TER-119, BD Pharmingen). For human vessel staining, a mouse monoclonal anti-human CD31 antibody (1:100, clone JC70A, Dako), detected by the avidin-biotin complex Kit (ABC System, SP-2002, Vector Laboratories) and developed with diaminobencidine staining (DAB plus chromogen solution, K3468, Dako) was used. Basal keratinocytes were detected by a mouse monoclonal anti-cytokeratin 14 antibody (4 ng/µl, clone LL001, Santa Cruz) followed by ABC and DAB detection and development. A rabbit monoclonal anti-Ki67 antibody (2 µg/mL, clone 30-9, 790-4286, Roche) was used to detect the proliferative cells. Hematoxylin and eosin (H&E) staining was done in a linear slide stainer (Leica ST4040) using Mayer's Hemalaun (1.09249.2500, Merck) and Eosin Y (1.15935.0100, Merck). For Masson-Goldner trichrome and elastica staining, kits were used according to the manufacturer's recommendations (12043, 14604, Morphisto). Collagen fibers in HE stained sections were stimulated with polarized light, using an Olympus^TM^ Rotatable Analyzer (U-AN360-3) filter. Human cell nuclei were labeled via in situ hybridization as previously published 46.

For immunofluorescence staining of paraffin embedded FSOs, a mouse monoclonal anti-human vimentin antibody (1.56 µg/mL, clone V9, M072501-2, Dako), a mouse monoclonal anti-human CD31 antibody (1:10, clone JC70A, Dako) and a mouse monoclonal anti-cytokeratin 14 antibody (40 ng/µl, clone LL001, Santa Cruz), a mouse monoclonal anti-cytokeratin 10 (K10, 1.2 µg/mL, AE20, ThermoFisher) and a mouse monoclonal anti-cytokeratin 1 (K1, 5 µg/mL, LHK1, ThermoFisher) were used, followed by secondary antibodies goat anti mouse IgG, Alexa Fluor 488 and 594 (A-11001, A-11005; Invitrogen). For co-staining sequential primary and secondary antibody treatment was applied to avoid unspecific binding. For immune-histochemistry of FSOs, a rabbit monoclonal anti-Ki67 antibody (2 µg/mL, clone 30-9, 790-4286, Roche) was used to detect the proliferative cells and an anti-PanK (28 µg/mL, EPR17341, Ventana) was used to detect the epidermal layers.

### Image Acquisition

Life cell imaging and video recording was performed on an Eclipse Ti inverted microscope (Nikon) with a customized live cell incubation system (Oko lab). Images were taken every 15 minutes for 48 hours. Image analysis was done using NIS elements imaging software AR 4.30.02 (Nikon). Confocal microscopy was performed using laser scanning microscopes Axio Observer Z1 attached to LSM700 (Carl Zeiss). Light microscopic cell culture pictures were generated with an EVOS XL microscope (Thermo Fisher). Total slides were scanned automatically in 40x magnification using the VS-120-L Olympus slide scanner 100-W system and processed using the Olympus VS-ASW-L100 program.

### Clonogenicity, EV concentration and Network formation assay

The clonogenic potential of fibroblasts, endothelial cells and hiPSC-derived cells was assessed according to published protocols 44,47. The purification of extracellular vesicles from hPL supporting the angiogenic potential of ECs and hiPSC-ECs as well as the network formation assay was previously established and performed according to a published protocol 48.

### Quantification, dermal scoring, statistical analysis

Vessels were quantified in dermal areas of 0.1 mm^2^ followed by counting the total number of vessels (murine + human vessels) in H&E stained sections using the Olympus VS-ASM-L100 program. The epidermal thickness was measured using the same program. Precise 4 µm sectioning of all skin grafts was done by the experts of the pathological institute at the University Hospital Salzburg using a microtome HM 355S (Thermo Scientific) including a section transfer system and providing consistent cutting conditions to ensure constant cutting angles in all grafts. The dermal score was evaluated to grade the dermal quality of the transplants, based on the density of fibroblasts, produced matrix, vessels, hemorrhage and collagen fibers produced. Areas of 0.1 mm^2^ were randomly selected and scored from 0 to 9 points (see **Table S3**). Per group, three to five biological and three technical replicates were included. Statistical analysis was performed using One-Way ANOVA analysis and multiple comparison in GraphPad Prism version 7.03. For proteome analysis background signals were subtracted and normalized compared to the reference spots locating on the arrays. Probes showing a normalization signal lower than 0.06 in the untreated samples were removed from analysis. Values were scaled using Z-score row scores and mapped on heatmaps using the R package “ComplexHeatmap” 49.

## Results

### Adult progenitor cell isolation, characterization and large scale expansion

**Figure 1:**
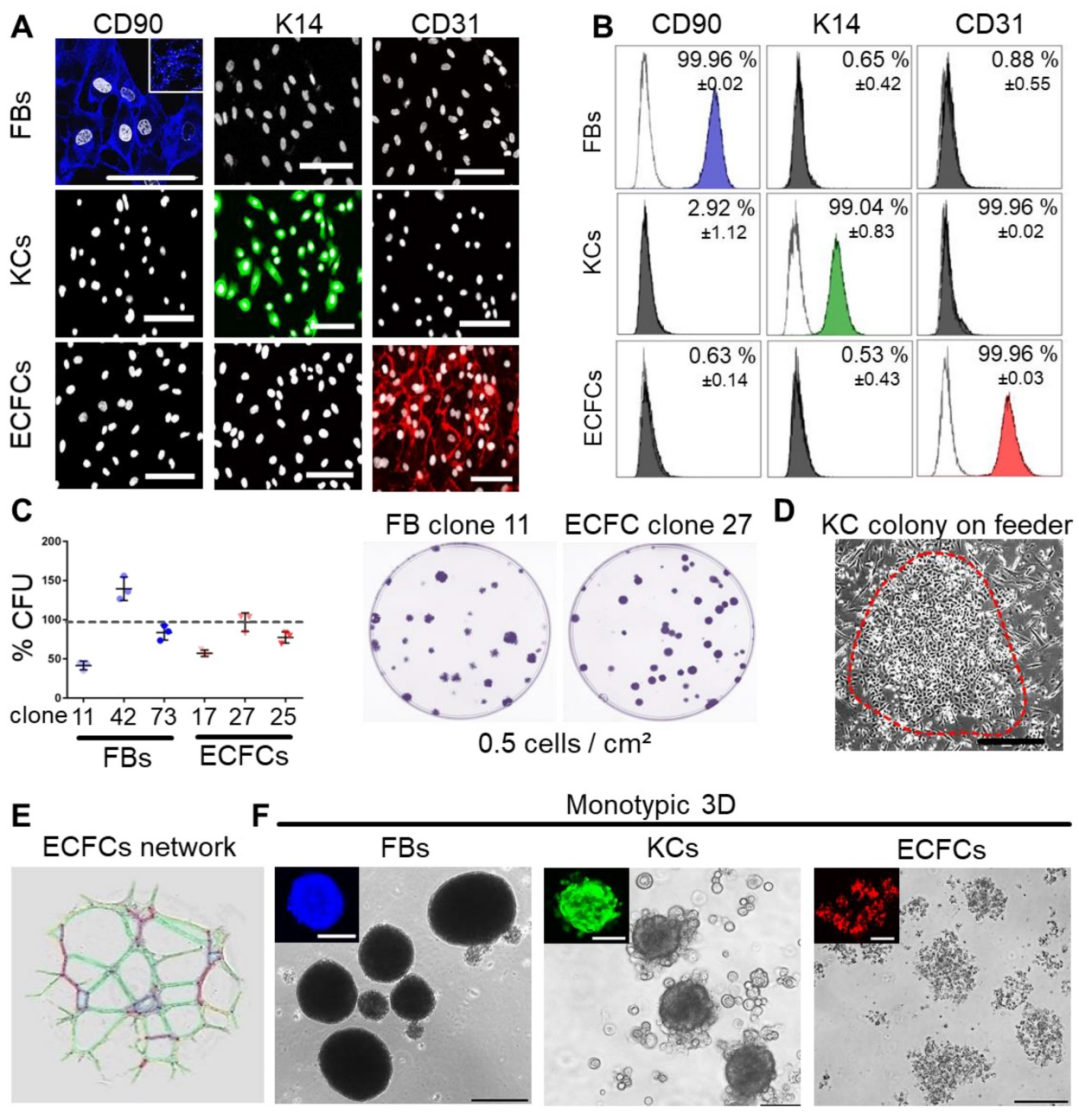
** Characterization of purified adult skin and vascular progenitor populations.** (**A**) Immune-fluorescence showing CD90 expression of culture-expanded adult skin fibroblasts (FBs, blue), intracellular keratin 14 (K14) expression of keratinocytes (KCs, green) and CD31 surface expression of endothelial colony-forming progenitor cells (ECFCs, red; all pseudo-colored). (**B**) Flow cytometry confirmed purity of isolated cells. Representative histograms shown; (n = 3 biological replicates; mean ± SD). (**C**) CFU assays showed donor-dependent clonogenicity of FBs and ECFCs (n = 3; mean ± SD). (**D**) KC colony on a feeder layer. (**E**) Vascular network formation after 12 hours on matrigel confirmed angiogenic potential of ECFCs (color-coded for automatic counting). (**F**) Primary skin FBs and KCs, but not ECFCs formed compact monotypic 3D spheroids (FBs = blue-, ECFCs = red-, KCs = green-labeled with nanoparticles). (**A, D-F**) Data from one of three biological replicates shown. Scale bar = 100 µm.

Progenitor cells from many other organs can assemble in spheroids when subjected to ultralow attachment environment. We tested this behavior for three skin-related cell types. Primary keratinocytes and fibroblasts were isolated from epidermal and dermal split-skin parts, respectively. Endothelial colony-forming progenitor cells (ECFCs) served as a universal source of high endothelial cell numbers based on previously established protocols after late outgrowth from umbilical cord blood 26,43,50. All cell types were large-scale expanded under animal serum-free conditions 39,51 (**Figure S1A**). Cell characterization showed specific CD90^+^ fibroblasts, keratin 14^+^ keratinocytes and CD31^+^ ECFCs (**Figure 1A-B**). Clonogenicity indicated progenitor enrichment after low seeding density propagation selecting for high proliferative potential cells 26,52,53 with donor-dependent high colony-forming unit (CFU) capacities in fibroblasts and ECFC preparations (**Figure 1C**). Keratinocytes also grew clonally on feeders after primary culture (**Figure 1D**) with donor variation in morphology and keratin 14 expression between 19.1 - 97.2% (**Figure S1B**). More than 90% pure keratin 14^+^ cultures, enriched for highly proliferative basal epidermal keratinocyte progenitors, were selected for subsequent organoid formation and transplantation experiments. ECFC functionality was confirmed by vascular network formation (**Figure 1E**). Both fibroblasts and keratinocytes regularly formed compact 3D spheroids. ECFCs formed 2D colonies (**Figure 1C**) and loosely accumulated in 3D (**Figure 1F**).

### Platelet-derived growth factors promote mixed progenitor cell self-assembly into organoids

**Figure 2:**
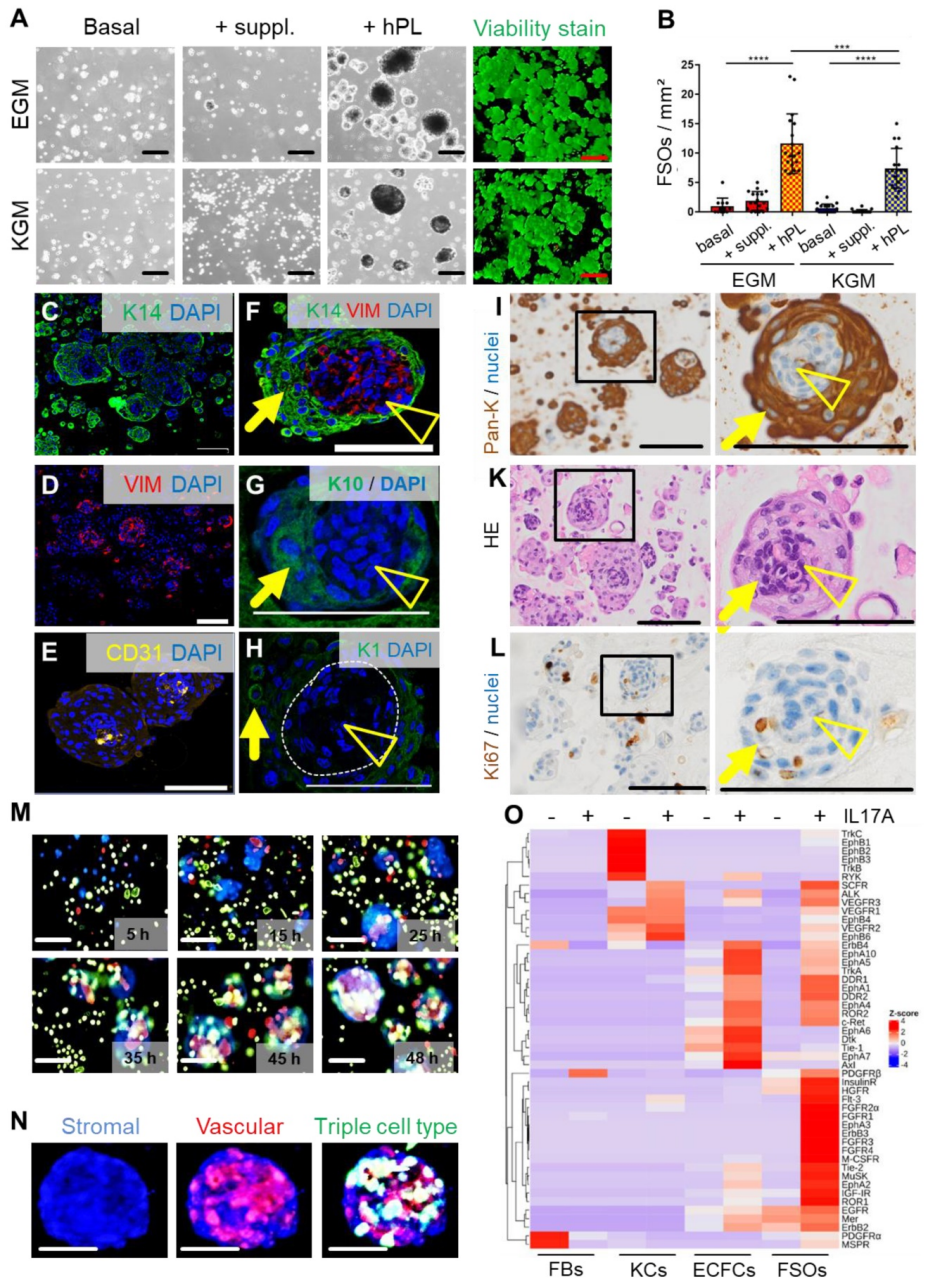
** 3D self-organization and FSO functionality.** (**A**) Triple cell type organization of fibroblasts, keratinocytes and endothelial cells as evaluated in endothelial growth medium (EGM) and serum-free keratinocyte growth medium (KGM). Light microscopy showed viable (green fluorescence viability dye-positive) FSO formation promoted by platelet-derived growth factors (+hPL). Basal media without hPL or with basic supplements (+suppl.) did not support proper 3D organization. (**B**) Significantly more FSOs were formed in the presence of hPL. One-Way-ANOVA and multiple comparison of three areas of two biological and three technical replicates; ***p<0.001, ****p<0.0001. (**C to L**) Histology of cross-sectioned organoids harvested at day 6. Anti-human keratin 14 (K14, green; **C**), anti-human vimentin (VIM, red; **D**) and anti-human CD31 (yellow; **E**) confirmed cell organization into dermal core structures (VIM + CD31) and epidermal layered arrangement (K14). (**F**) K14 / VIM, (**G**) and K10, (**H**) K1; DAPI^+^ nuclei in blue. (**I**) Pan-keratin staining of the epidermal cell layers. (**K**) Hematoxylin/eosin staining illustrated FSO organization. (**L**) Anti-Ki67 staining indicating cell proliferation within FSOs. (**I-L**) Boxes indicating area shown in higher magnification at the right next to overview pictures. Arrowhead = dermal core; arrow = epidermal surface. (**M**) Still pictures with time insert from live cell-tracking the 3D organization process with fluorescent nanoparticles (fibroblasts, blue; endothelia; red; keratinocytes, green; see Supplementary Movie 1). FSO 3D assembly starting from initial stromal-vascular aggregation and followed by superficial anchorage of adult KCs, indicating well-organized FSOs at app. 48 h after cell seeding, repeated in triplicates. (N) Representative higher resolution image of a 6-day assembled organoid, labelled with fluorescent nanoparticles nanoparticles (fibroblasts, blue; endothelia; red; keratinocytes, green). (**A and C to N**) Organoid preparation was done using three independent donor combinations (3 biological replicates). Scale bar = 100 µm. (**O**) Proteome profiling of un-starved single FBs, ECFCs, KCs and 4-day assembled organoids after 12 h stimulation in the absence (HCL control) or presence of IL17A. Z-scores (selected representative analysis; see also Supplementary Fig. 2).

Spheroid formation by purified expanded adult fibroblasts, keratinocytes and endothelial cells prompted us to ask the question if it is also possible to instantly create spheroid skin organoids composed of all three cell types using contemporary technology as an alternative to protracted conventional skin equivalent protocols. FSOs were established by proportional seeding of keratinocyte, fibroblast and endothelial single-cell suspensions (2:1:1) in appropriate media. Different serum-free and serum-supplemented media supported organoid formation, which significantly increased in the presence of hPL as a source of a complex mixture of natural human platelet-derived growth and regenerative factors (**Figure S2A, Figure 2A-B**). Histology of paraffin-embedded FSO cross sections using specific keratin 14, pan-keratin, CD31 and vimentin labelling, revealed a compact dermal core structure, covered by compactly arranged epidermal layers (**Figure 2C-I**). Anti-CD31 staining showed endothelial clusters properly located within the dermal core (**Figure 2E**). Keratin 10 and keratin 1 expressing cells were detected within the epidermal layers, indicating keratinocyte differentiation at day 6 (**Figure 2G-H**). Pan-keratin staining illustrated the multilayered epithelium surrounding a stromal core (**Figure 2I**). Hematoxylin and eosin staining confirmed the FSO structure composition (**Figure 2K**). Proliferation within the epidermal compartment was evidenced by Ki67 staining (**Figure 2L**). Using fluorescent nanotracker-labeled cells we detected fibroblast aggregation within <10 hours after seeding, followed by stromal-vascular dermal-like core formation, and keratinocytes superficially settled after 24 hours. Video-imaging visualized consecutive FSO assembly, followed by ECFC arrangement and surface anchorage of KCs (**Movie S1, Figure 2M-N**).

To assess functionality of FSOs as in vitro model of multicellular skin-like structures, we subjected FSOs as well as their constituting single cell types to kinase-dependent skin inflammatory IL-17A input signals. Kinome profiling using three different antibody array signatures (n = 11 arrays) constantly showed time-dependent significant differences in organoid response patterns compared to the respective single cell types. Baseline platelet-derived growth factor (PDGF) and ephrin responses to cell culture conditions were prominently detectable in non-starved fibroblasts and keratinocytes, respectively, with the latter as well as ECFCs properly responding to IL-17A. The baseline signaling vanished under FSO conditions as Z-scoring accentuated the complex IL-17A response pattern in the 3D structures. Time course confirmed more sustained phosphorylation responses in FSOs compared to the usually hyper-acute signaling in single cells (**Figure 2O, Figure S2B-C, E**).

### Self-organization of human single-cell suspension transplants creating neo-vascularized planar human skin

**Figure 3:**
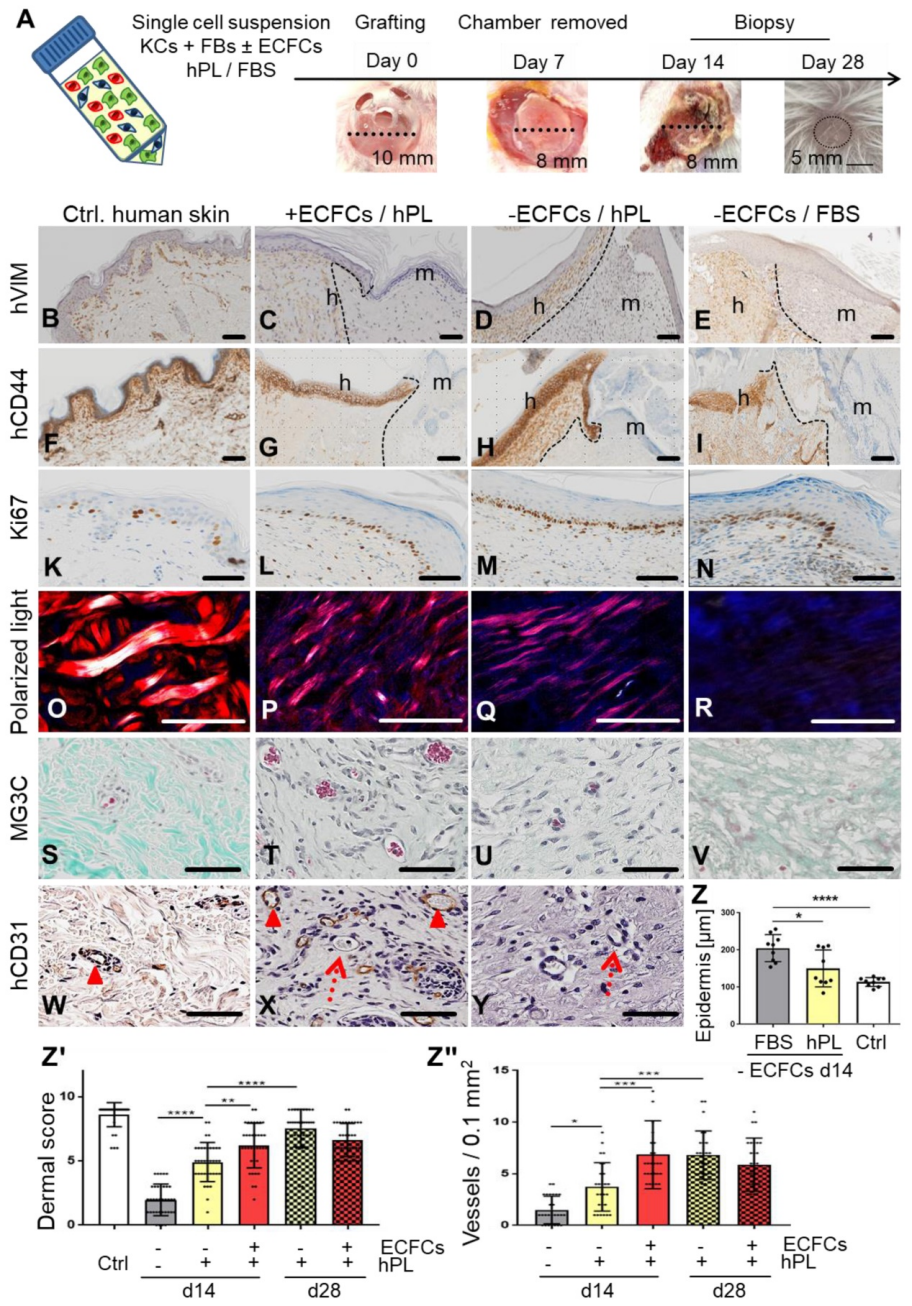
** Skin cell self-organization and endothelial cell co-transplantation resulting in properly layered and rapidly vascularized human skin in vivo.** (**A**) Grafting procedure illustration: single-cell suspensions (KCs + FBs ± ECFCs) in 10% hPL- or FBS-supplemented medium were transplanted in a silicone chamber inserted in an eight mm full-thickness wound of NSG mice. Chambers were removed at day 7 and grafts analyzed days 14 or 28. (**B to Y**) Histology of transplants in the absence or presence of ECFCs, supported by hPL or FBS, compared to (**B and F and K and O and S and W**) healthy human control skin. (**B to I**) Anti-human vimentin (hVIM, brown, **B to E**) and anti-human CD44 staining (brown, **F to I**) confirmed the human origin of the epidermis and the dermis, showing the borders between human and murine skin (black, dashed lines), and stratified skin organization. h = human; m = murine. (**K to N**) Anti-Ki67-labelled proliferating cells (brown). (**O to R**) Polarized light-activated collagen fibers exclusively in control skin and hPL-supported, not in FBS-driven transplants (**S to V**). Masson-Goldner trichrome (MG3C) histochemistry showed vessel enrichment when ECFCs were co-transplanted with fibroblasts/keratinocytes in the presence of hPL (+ECFCs/hPL) compared to transplants without ECFCs (-ECFCs/hPL) showing occasional murine vessel sprouting. Murine erythrocytes (red) confirmed blood circulation inside vessels. (**W to Y**) Anti-human CD31 verified human vessel origin in +ECFCs/hPL. Dotted red arrows = murine vessels. Filled arrowheads = human vessels. (**B to Y**) Scale bar = 100 µm. One out of three to five independent grafts per group shown. (**Z**) Quantification showing significantly increased epidermal thickness in -ECFCs/FBS compared to -ECFCs/hPL. (**Zꞌ**) Dermal quality score was significantly increased in hPL- compared to FBS-supported transplants and by ECFC presence day 14 after transplantation. (**Zꞌꞌ**) Vessel number in grafted cell-derived self-organized human dermis was significantly increased day 14 after ECFC co-transplantation. (**Z to Zꞌꞌ**) One-Way-ANOVA, multiple comparison of three biological and three technical replicates; *p<0.05, **p<0.01, ***p<0.001, ****p<0.0001.

We next tested if self-assembly of primary human skin cell suspensions in the absence or presence of ECFCs and platelet-derived factors, resulted in vascularized human skin formation. Xenotransplantation was performed after inserting a silicone grafting chamber into full-thickness eight mm diameter skin wounds of immune-deficient NSG mice. Single-cell suspensions of fibroblasts and keratinocytes were either pooled in fetal bovine serum (FBS) as a control as described previously 45, or in 10% hPL-supplemented media. ECFCs were added as indicated immediately prior to grafting the cell suspension onto the murine muscle fascia inside the grafting chamber. Chambers were removed seven days after grafting, allowing the human graft to connect with the murine wound edges (**Figure 3A**). Skin biopsies taken 14 and 28 days post grafting revealed cell organization into human epidermal and dermal layers (**Figure 3C-E, G-I, Figure S3, Figure S4, Figure S5A-B**). Histology confirmed human cell origin and appropriate epidermal and dermal cell location in the grafts (CD44^+^, human epidermal and dermal cells; vimentin^+^, human dermal cells; K14^+^, human epidermal cells; EGFR^+^, human epidermal cells) (**Figure 3B-I, Figure S3, Figure S4, Figure S5B**). Proliferating cells were found restricted to basal epidermal keratinocyte layers and scattered dermal cells (**Figure 3L-N**) comparable to human abdominal wall control skin (**Figure 3K**).

Based on our observation that platelet-derived factors supported progenitor self-assembly into skin organoids in vitro, we next tested different grafting concentrations of hPL (1%, 10%, 100%) and found consistent epidermal-dermal composition with limited erythrocyte extravasation preferentially with 10% hPL-supplemented transplant medium (**Figure S5F**). Histology quantification showed significant epidermal thickening with signs of hyperkeratosis in FBS-containing transplants reminiscent of inflammatory responses. Epidermis created under the aegis of hPL did not differ significantly in thickness from healthy human abdominal control skin (**Figure 3Z**). Dermal scoring (**Table S1**) was significantly higher in hPL- compared to FBS-containing transplants indicating superior dermal organization with hPL (**Figure 3Zꞌ**). Moreover, 28-day skin cell grafts in hPL resulted in more mature dermal architecture as evidenced by significantly increased dermal score compared to 14-day grafts (**Figure 3Zꞌ**), comparable to normal skin (**Figure S5C-D**). The presence of hPL supported normal epidermal development, normal dermal cell distribution, collagen production and murine sprouting angiogenesis compared to FBS. This was shown by hematoxylin/eosin, elastica staining (**Figure S5C, Figure S5D**), polarized light stimulation (**Figure 3O-R**) and trichrome histochemistry particularly in the presence of ECFCs and hPL (**Figure 3S-V**). Successful tissue regeneration depends on effective vascularization of injured organs, rapidly supporting the tissue with oxygen and nutrients 22. Extending previous experience with robust human vasculogenesis induced by combining ECFCs with stromal cells 26, co-transplantation of ECFCs significantly increased dermal vessel formation compared to keratinocyte/fibroblast-transplants devoid of ECFCs, despite adding hPL in both conditions (**Figure 3Zꞌꞌꞌ, Figure 3T**). De-novo formed CD31^+^ human vessels co-localized with adjacent in-grown presumably murine vessels lacking CD31 (**Figure 3X**). Abundance of murine erythrocytes further indicated proper blood circulation and connection of human with murine vasculature, particularly in hPL-supported transplants (**Figure 3T-U**). A summary of transplant groups and key test parameters is given in **Table S2**.

### Sustainable ectodermal lineage specification of hiPSC-KCs augmented by fibroblast growth factor-7 mRNA transfer

**Figure 4:**
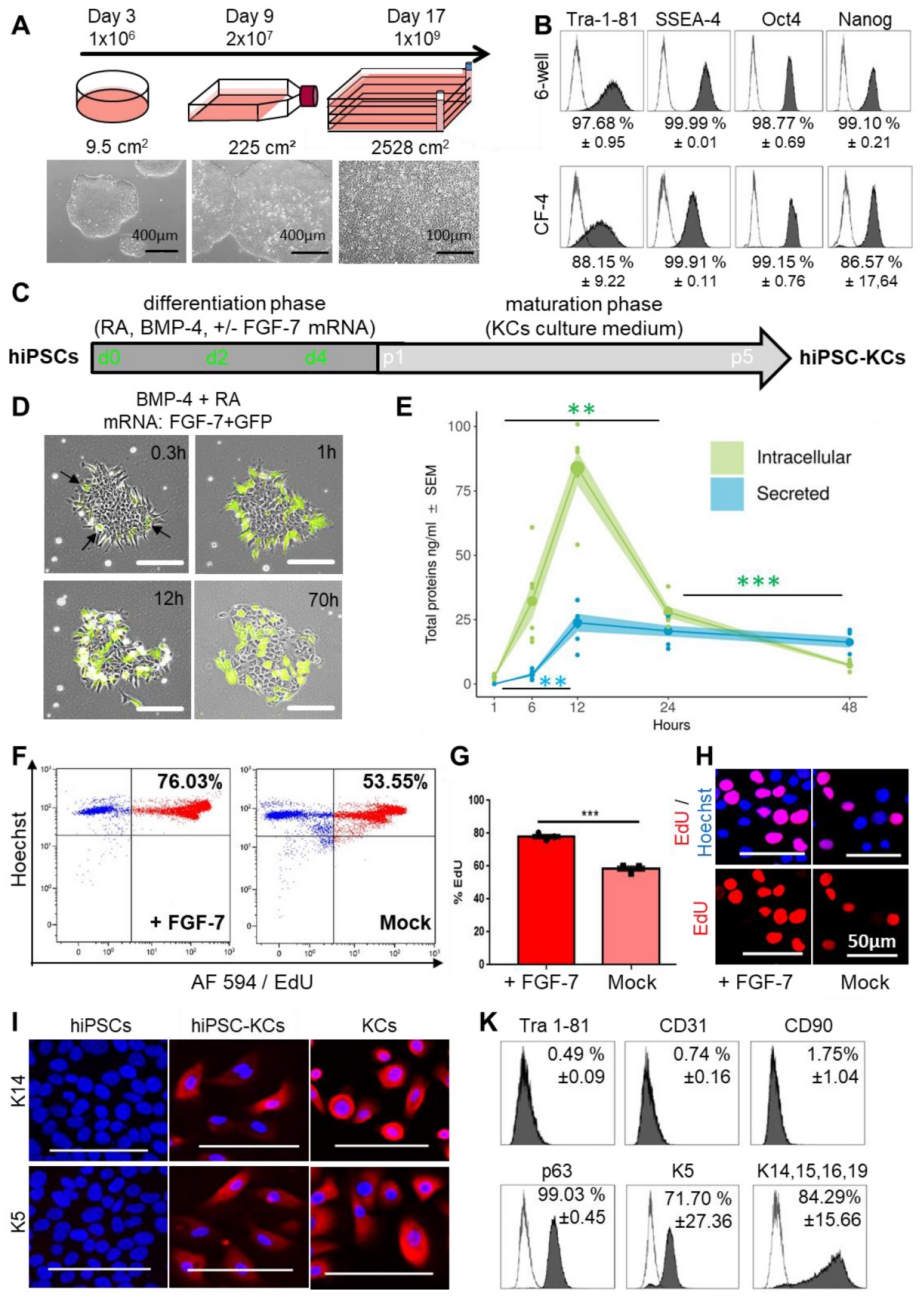
** FGF-7 mRNA transfection improved ectodermal cell specification from hiPSCs. (A)** hiPSC colonies were thawed at day 0 and seeded in a 6-well plate. At day 3 the cells were harvested at 80% confluency (≈1 x 10^6^ cells) and transferred into a T225 flask. At day 9 cells reaching 80% confluency (≈20 x 10^6^ cells) were transferred into a 2,528 cm^2^ cell factory (CF-4). The cells continued to grow clonally and were harvested at day 17 as single cells (≈1 x 10^9^ cells). **(B)** Marker expression (Tra 1-81, SSEA-4, Oct4 and Nanog) of hiPSCs at day 3 and day 17 revealed maintenance of pluripotency upon large-scale expansion (one representative histogram of two independent clones; mean ± SD). (**C**) Schematic depiction of the keratinocyte differentiation protocol from hiPSCs. (**D**) Stabilized FGF-7 and GFP mRNA co-transfection indicated protein expression starting 18 minutes after transfection by green fluorescence. (**E**) FGF-7 ELISA confirmed significantly increasing intracellular (green) FGF-7 protein expression over time with maximum intracellular expression levels of 83.8 ± 6.5 ng/ml 12 h after transfection (green). FGF-7 protein secretion (blue) was lower compared to intracellular levels 12 h after transfection (triplicate transfections of n = 2 independent clones, duplicate analysis; individual data points as filled small circles and mean values as large circles connected by lines, ±SEM shaded areas). **p<0.01, ***p<0.001. (**F and G**) Flow cytometry (representative histograms in **F**) of 5-ethynyl-2'-deoxyuridine (EdU)-labelled hiPSC-KCs during the maturation phase showing significantly increased proliferation rate of hiPSC-KCs + FGF-7 mRNA compared to Mock transfected cells. Unpaired t test of three independent experiments. (**H**) Fluorescence microscopy confirmed the EdU incorporation into nuclei. (**I**) Immunofluorescence staining demonstrated keratin (K5/K14) expression in mature basal progenitor-type hiPSC-KCs + FGF-7. (**K**) Flow cytometry showing Tra 1-81^-^/CD90^-^/CD31^-^, p63^+^, keratin K5^+^/K14^+^/K15^+^/K16^+^/K19^+^ hiPSC-KCs population at passage four (one representative histogram of two independent hiPSC clones; mean ± SD).

We next aimed to build hiPSC-derived skin cells for testing their self-organization capacity. Human iPSCs represent a versatile source for differentiated cell propagation for experimental and transplant purposes, particularly in case of limited tissue availability, e.g. after extensive skin burns 54. The hiPSC clones were first large-scale expanded, generating one billion cells per batch from less than one million starting cells within 17 days (**Figure 4A**) while maintaining a pluripotency phenotype (**Figure 4B**)**.** Three-lineage differentiation with improved protocols was performed in parallel for subsequently testing self-organization of hiPSC-derived keratinocytes (hiPSC-KCs), fibroblasts (hiPSC-FBs) and endothelial cells (hiPSC-ECs). Keratinocytes as ectodermal lineage progeny were differentiated by combining retinoic acid and bone morphogenetic protein as described 41, with moderate differentiation (**Figure S6A**), but limited proliferation upon maturation beyond passage three (**Figure S6C**). We thus hypothesized that instant availability of a keratinocyte growth and survival factor could enhance differentiation and proliferation for producing sufficient numbers of functional hiPSC-KCs for further testing. Based on clinical experience with kepivance, we selected fibroblast growth factor-7 (FGF-7; also known as keratinocyte growth factor, KGF) that is otherwise provided by fibroblasts and intra-epithelial γδ T cells in vivo 13,55. The hiPSCs were transfected based on our recently established protocol 38 with stabilized FGF-7 mRNA during early differentiation (**Figure 4C**). Transfecting hiPSCs with green fluorescent protein (GFP) mRNA as a reporter revealed >40% transfection efficiency 24 hours post transfection (**Figure S6B**). Combined transfection of FGF-7 and GFP mRNAs indicated protein expression starting 18 minutes after transfection by green fluorescence (**Figure 4D, Movie S2**). Appearance of hiPSC clones changed immediately after initiating ectoderm specification into epithelial-like structures (**Figure 4D, Movie S3**)**.** Intracellular FGF-7 protein expression reached a maximum after 12 hours of mean 83.8 ± 6.5 ng/ml. Mean 23.8 ± 3.5 ng/ml FGF-7 protein was secreted 12 hours after transfection (**Figure 4E**). The mRNA-based intervention resulted in significant increased proliferation rates of FGF-7-nurtured hiPSC-KCs compared to Mock transfected cells as evidenced by significantly increased deoxyuridine uptake in flow cytometry and nuclear localization deoxyuridine signals (**Figure 4F-H**). Further maturation of hiPSC-KCs after FGF-7 transfection resulted in successive epithelial morphology acquisition, persistent extensive proliferation beyond passage four (**Figure S6C**) and specific keratin (K5, K14) expression (**Figure 4I**)**.** Flow cytometry confirmed loss of pluripotency markers with assuming a mature hiPSC-KC phenotype comparable with basal KC progenitors (**Figure 4K, Figure S7A**)**.**

### Platelet-derived factors promote mesodermal specification of hiPSCs and ameliorate angiogenic potential of hiPSC-derived endothelial cells

**Figure 5:**
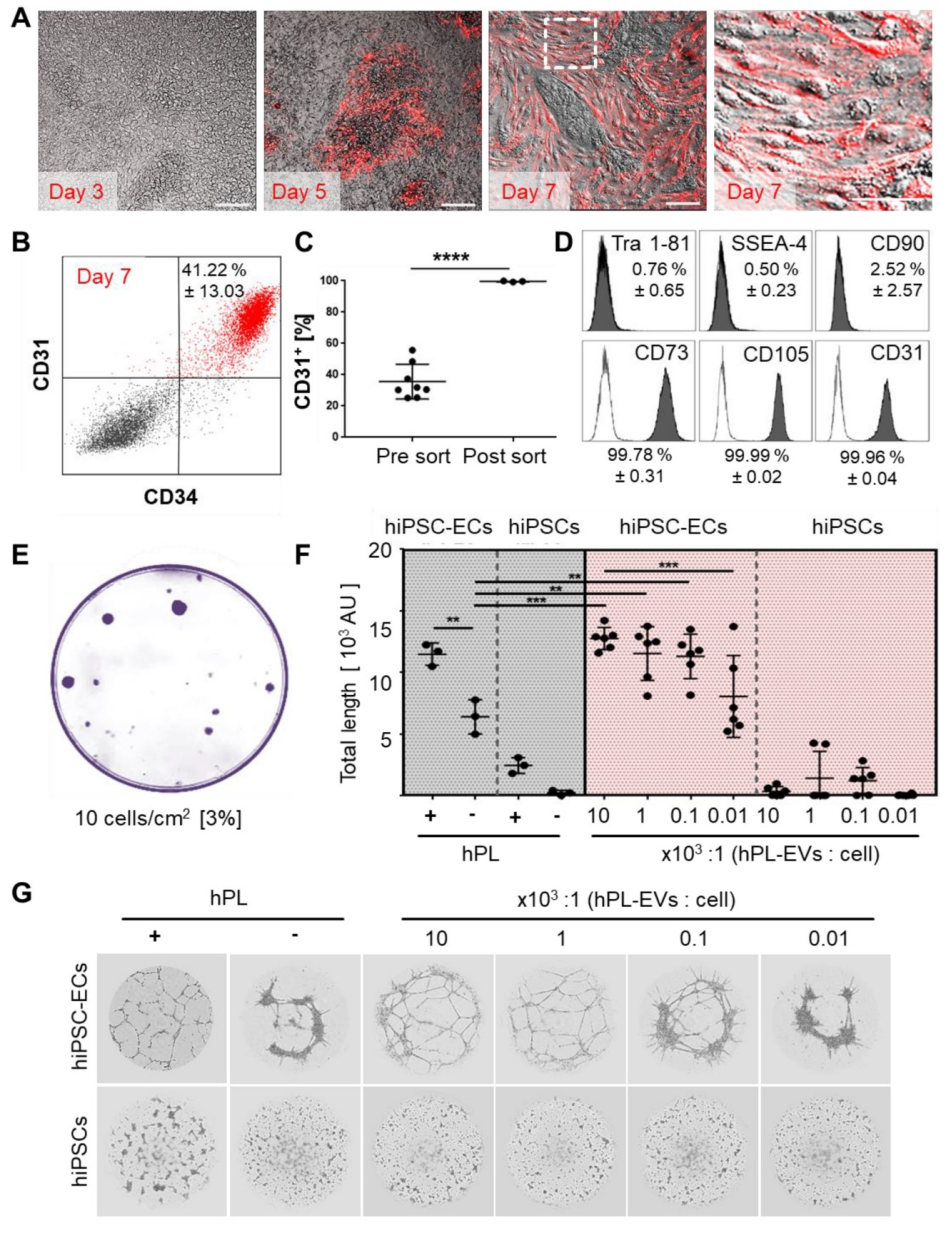
** Platelet-derived extracellular vesicles augment angiogenic function of hiPSC-ECs.** (**A and B**) Repetitive in situ reporter staining of hiPSC-EC cultures during differentiation using an anti-human CD31 antibody highlighting differentiated cells (red), corresponding to (**B**) 41.22 ± 13.03% CD31^+^/CD34^+^ hiPSC-ECs in flow cytometry. One representative histogram of three independent hiPSC clones; mean ± SD. (**C**) Differentiation efficiency towards CD31^+^ hiPSC-ECs was mean 35.6 ± 3.9 % (n=8), and sort-purification led to 99.7 ± 0.2 % (n = 3). Statistical analysis in GraphPad Prism 7.03, t-test, 3 independent hiPSC clones; **** p<0.0001. (**D**) Phenotypic analysis showing pure Tra 1-81^-^, SSEA-4^-^, CD90^-^, CD73^+^, CD105^+^, CD31^+^ mature hiPSC-ECs. Representative histograms of two independent hiPSC clones shown; mean ± SD. (**E**) Colony-forming unit assay (CFU) showed 3% clonogenic potential of hiPSC-ECs seeded at a density of 10 cells/cm^2^. Representative example shown, n = 3. (**F**) Vascular-like network formation quantification of total network length showing angiogenesis by differentiated hiPSC-ECs, but not hiPSCs; platelet-derived growth factor (hPL)-dependent; increased network formation with increasing hPL-derived extracellular vesicle (EV) dose. One-Way-ANOVA and multiple comparison of two independent hiPSC donors, repeated in triplicates; **p<0.01, ***p<0.001. (**G**) Vascular-like networks formed on matrix. Representative examples shown, n = 6.

Aiming to derive dermal hiPSC-FB, mesoderm induction, stromal specification and maturation was performed according to our hPL-based protocol 37 (**Figure S7A, Figure S8A-B**). Mature hiPSC-FBs were rich in clonogenic progenitors (**Figure S8C**) and could be propagated in 3D (**Figure S8D**)**.** CD26 (dipeptidyl peptidase-4) expression of mature hiPSC-FBs ranged from 20% to 50%, reminiscent of a neonatal fibroblast phenotype 56 (**Figure S7B-C**). For transplant vascularization, hiPSC-ECs were generated based on published protocols 42 with modifications (**Figure S9A**). In situ reporter staining using CD31 antibodies allowed non-invasive online monitoring of hiPSC-EC appearance and maturation in culture, revealing time-dependent increase to up to 41.22 ± 13.03% CD31^+^/CD34^+^ hiPSC-ECs until day seven (**Figure 5A-B**).

Mean differentiation efficiency was 35.6 ± 3.9% CD31^+^ cells before and 99.7 ± 0.2% CD31^+^ after cell sorting (**Figure 5C, Figure S9B**) with a mature Tra 1-81^-^, SSEA^-^, CD90^-^ and CD73^+^, CD105^+^, CD31^+^ EC phenotype (**Figure 5D, Figure S7A**) and clonogenic potential (**Figure 5E**). In this study, we did not test more extensively whether bona fide ECFCs were initiating the colonies 31,57 because the focus was on using post-natal and hiPSC-derived endothelia for organ and organoid vascularization. Purified hiPSC-ECs formed pronounced vascular networks in the presence but not absence of hPL. To gain further insight into the mechanism of hPL-based angiogenesis support 26,30 we purified extracellular vesicles (EVs) from hPL, because we observed in another study that EVs can mediate such trophic effects 48. The hPL-derived presumably platelet lipoprotein-enriched EVs 48 supported angiogenesis of hiPSC-ECs significantly in a dose-dependent manner even in the absence of otherwise essential externally added vascular endothelial growth factor (VEGF). Undifferentiated hiPSCs did not respond to pro-angiogenic stimuli (**Figure 5F, Figure 5G**).

### Self-assembly of hiPSC-derived FSOs in vitro and of single-cell suspension transplants in vivo

**Figure 6:**
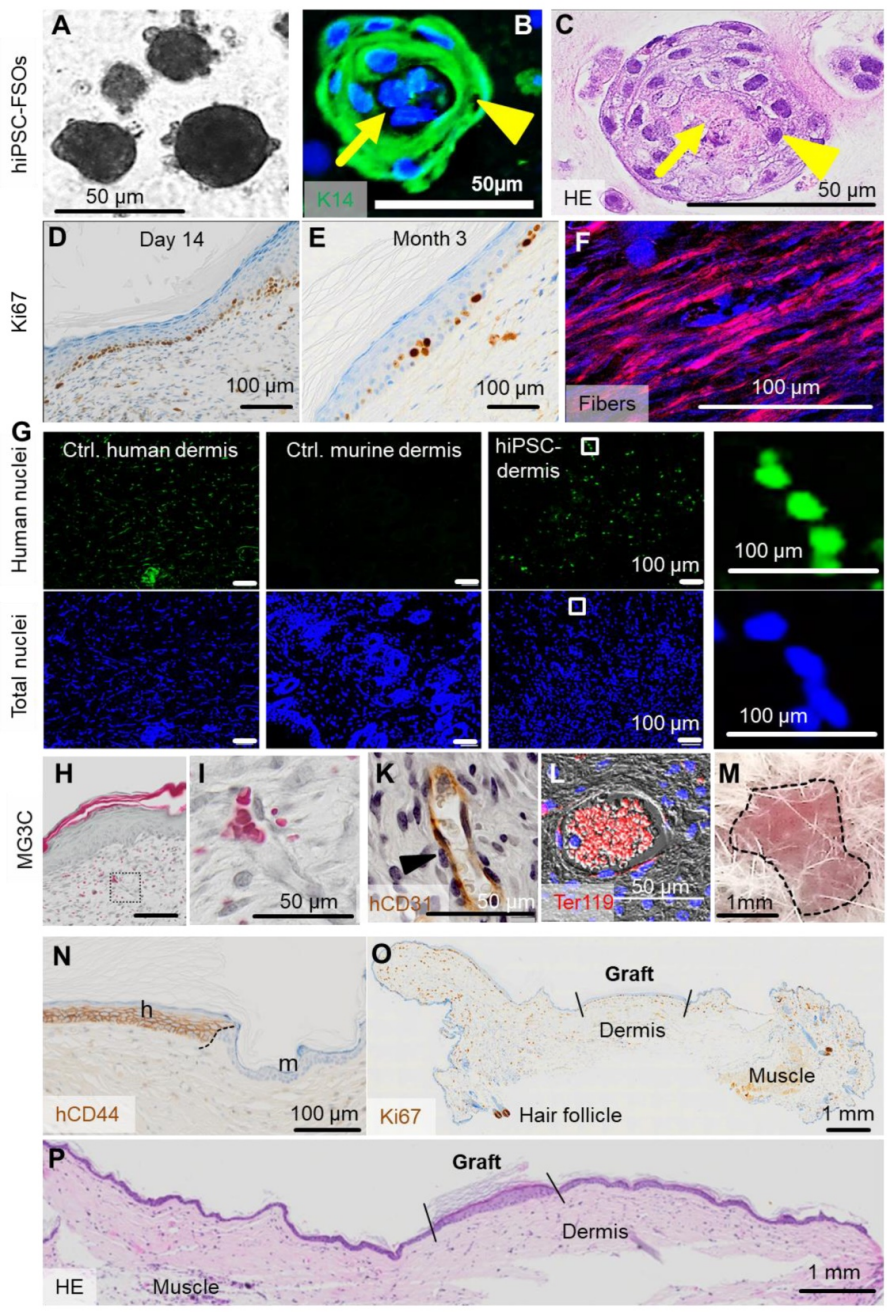
**(previous page): In vivo self-assembly of hiPSC-derived human skin.** (**A**) Triple cell type organization of hiPSC-FBs, hiPSC-KCs and hiPSC-ECs in EGM + hPL resulted in hiPSC-derived FSOs in vitro. (**B and C**) Histology of hiPSC-FSOs showed a dermal core structure (arrow) with epidermal surrounding layers (arrowhead), determined by K14 epidermal cell labelling (**B**) and hematoxylin and eosin staining (**C**). (**A to C**) Organoid assembly was performed using cell differentiated from two independent hiPSC clones. (**D**) Single-cell suspension transplants of hiPSC-derived cells were analyzed day 14 and (**E**) three month after transplantation. Histology confirmed epidermal-dermal stratification showing proliferating Ki67^+^ basal keratinocytes. (**F**) Polarized light stimulation confirmed maturing collagen fibers day 14. (**G**) In situ hybridization marked human nuclei (Alu sequences, green fluorescence) at day 14. DAPI labelling of total human and murine cell nuclei in blue. (**H and I**) Masson-Goldner trichrome (MG3C) staining showed a vascularized dermis, (**I**) higher magnification, day 14. (**K**) Anti-human CD31 staining labelled hiPSC-ECs along the vessel wall (brown) stabilized by pericytes (blue nuclei in unstained adjacent cells, arrowhead). (**L**) Anti-murine glycophorin-related Ter119 staining confirmed blood supply within vessels (red fluorescence). Scale bar 100 µm. (**M**) Self-assembled hiPSC-derived human skin showed no macroscopic signs of tumor formation three months after grafting (representative picture; n = 2 animals). (**N**) Anti-human CD44 staining confirmed the human origin of the skin graft, showing the border (black dashed line) between human and murine skin. h = human, m = murine. (**O**) Anti-Ki67, although not human-specific, revealed no aberrant high-proliferative structures three months after grafting (panniculus carnosus, murine muscle; murine hair follicle). (**P**) Hematoxylin/eosin staining confirmed the absence of tumorigenic structures in serial full section scans three months after grafting (A to L). Scale bar=100 µm. n = 2 animals per condition.

To test the self-organization of hiPSC-derived skin cells into organoids in vitro, ectodermal hiPSC-KCs and mesoderm-derived hiPSC-FBs plus hiPSC-ECs were combined (ratio 2:1:1) as established before in this study for adult progenitors (**Figure 6A-C**). To question self-organization in vivo, single-cell suspension transplants consisting of these three hiPSC-derived cell types in 10% hPL-supplemented transplant medium were prepared as established before with adult cells and grafted into eight mm diameter full-thickness skin wounds on the back of NSG mice**.** Analysis was performed 14 days and three months after transplantation (**Figure 6D-P**)**.** Histology confirmed proper organization into epidermal and dermal layers with proliferating Ki67^+^ hiPSC-derived epidermal cells predominantly in the basal KC layer, still detectable after three months (**Figure 6D-E**). Polarized light microscopy 58 highlighted maturing collagen fibers produced by hiPSC-FBs (**Figure 6F**). In situ labelling of human nuclei using arthrobacter luteus (Alu) repetitive sequences 46 confirmed the human origin of dermal cells (**Figure 6G**). The human epithelial origin was confirmed using anti-human CD44 and anti-human EGFR staining, marking the borders between human and murine tissue (**Figure 6N, Figure S4**). Histochemistry showed vascularized hiPSC-derived dermis and perfused vasculature after two weeks, confirmed by human-specific CD31 staining and filled with murine erythrocytes (**Figure 6H-L**).

Contamination of transplants with immature hiPSCs bears teratoma risk. We did not observe teratoma formation within a 3-month observation period in two animals tested, matching requirements previously summarized to minimize tumorigenic risk qualitatively 59. Three months after transplantation, macroscopic transplant analysis revealed no aberrant structures at the transplant site (**Figure 6M, Table S3**). Histology and Ki67 staining of the graft and the surrounding murine tissue showed skin wound contraction as typically observed in rodents, morphologically appropriate human epidermis and dermis, but no signs of malignancy and no highly proliferative Ki67^+^ structures in the human graft and the murine skin (**Figure 6N-P**).

## Discussion

Taking advantage of a robust self-assembly potential of progenitor-enriched skin fibroblasts and keratinocytes together with endothelial progenitors, we introduced a novel simplified of skin organoid. Pre-vascularized FSOs comprise keratinocyte multilayers anchored on a dermal core made of fibroblasts and endothelial cells. Self-organization of these FSOs was dependent on adding a complex mixture of human platelet-derived factors including extracellular vesicles. The rapid and scalable FSO assembly from either adult or hiPSC-derived progenitors represents an attractive innovation particularly for individualized and cell type-specific pharmaceutical testing. The differential response of FSOs to IL-17A and forskolin stimulation represents preliminary evidence but demands additional high throughput screening endeavors comparing conventional planar skin organoids with 3D single-cell-type spheroids and FSOs under multiple standardized conditions. Translating the cell self-organization approach into a technically straightforward 'liquid' transplantation protocol further allowed rapid vascularized full-thickness skin wound healing by adult skin cell suspensions as well as by hiPSC-derived progenitor-enriched skin cells. Significantly increased skin quality was observed upon engraftment in hPL compared to animal serum. We speculate that transplantation of adult or hiPSC-derived cell suspensions, by using their self-organization potential, opens versatile opportunities for developing novel cell therapy strategies not restricted to skin regeneration. The comparison to conventional planar skin organoids was beyond the scope of this study.

From a technology point of view, our study offers additional timely application paths: (i) Regarding adult cell-derived spray-on skin 60, addition of hPL to transplanted cell suspensions might offer the opportunity to better repair wounds with small amounts of regional cells. Mechanistically, HPL-EVs mediated this effect. (ii) Transfection of stabilized lineage-specific growth factor mRNA 61, in our study coding for the keratinocyte growth factor FGF-7 to improve keratinocyte long-term fitness, may be extended to other cell lineages refining hiPSC-derived cell transplantation strategies. (iii) Two types of reporter assays were tested in our study. Co-transfection of fluorescent protein mRNA (GFP) enabled indirect monitoring of KGF expression as confirmed by protein ELISA. Repetitive addition of fluorescent anti-CD31 antibodies allowed for monitoring endothelial cell maturation. Both strategies are expected to find their way into daily laboratory routine due to their simplicity and reproducibility. (iv) We provided preliminary evidence that FSOs created within 3 - 6 days continue to proliferate and display an organotypic molecular response not observed in the separated skin cell types. More complex but still precisely defined progenitor cell input into rapidly self-assembled skin organoids will also enable high throughput pharmaceutical and molecular testing 36.

The precise selection of contributing mature cell types is an advantage regarding safety of hiPSC-based skin cell transplantation but also represents the major limitation of the current study, because it is lacking hair and skin appendages. In a recent landmark study, complex organoid-like structures containing skin with hair and appendages were generated from hiPSCs with a step-wise protocol. Contaminating cartilage and other extracutaneous fetal tissue may currently limit applicability 13. The perfect cell-engineered skin transplant will also contain regionalized appendages, nerves and loco-typical hair composition. Combining self-assembly strategies with sophisticated developmental biology and cell sorting tools has the potential to realize such complex endeavours in the near future. Clinical application without immune suppression will depend on either using autologous cells or histocompatible iPSC sources. Another limitation of our study is the lack of mechanistic explanations for the cell self-assembly in the presence of hPL that was not observed before 30,51,62-65. The inflammatory caspase-4 was demonstrated to mediate vascular regeneration in the presence of hPL 66. Preliminary data in the current study showed that platelet-derived EVs could mediate the pro-angiogenic effects of hPL. It was previously shown that needle-free injection of 3D fibroblast spheroid-derived EVs ameliorated skin photoaging 67. Sphere-forming capacity was identified as an enrichment strategy for epithelial-like stem cells from equine skin 68. It was also shown recently that self-assembling 3D spheroid cultures of human neonatal keratinocytes have enhanced regenerative properties 69. Additional evidence in a separate study indicated that EVs can protect selected lipoproteins in their corona 48 thus focussing our ongoing search for key mediators of hPL-derived EV-mediated self-assembly and regeneration.

The FSO is characterized by its simplicity, reproducibility and scalability. In a related model system, 3D skin organoids made of fibroblasts and the keratinocyte cell line HACAT were successfully used to study melanoma cell line invasion and drug response testing 70. Our study used healthy adult and hiPSC-derived progenitors focussing on utilization of self-organization in non-malignant conditions including high content drug testing and regenerative therapies. Spontaneous dermal cell aggregation triggering mechanosensitive β-catenin activation in adjacent epidermal cells was recently discovered in an elegant study of avian skin development 71. Sphere formation was could represent a strategy to increase the ability of cultured human dermal papilla cells to induce hair follicles from mouse epidermal cells in 72. Recent landmark studies induced haired murine and human skin organoids as models for skin development from pluripotent cells 11-13. We hope that future cell-based skin regeneration strategies will create body region-specific integument with appendages including hair, sweat glands and sebacous glands as required.

## Conclusion

In summary, the current study demonstrates that self-assembly of primary and hiPSC-derived keratinocytes, fibroblasts and endothelial cells can generate instantly available spheroid skin organoids for high content screening and drug testing. Triple cell type suspension grafting into full thickness wounds created layered human skin containing de novo engineered human blood vessels that were connected with the murine acceptor vasculature in immunodeficient mice. Disposing this self-organization principle of stem/progenitor cells will help to develop novel skin regeneration and wound healing strategies.

## Supplementary Material

Supplementary figures and tables.

Supplementary movie 1.

Supplementary movie 2.

Supplementary movie 3.

## Abbreviations

Alu: arthrobacter luteus
Dapi: 4′,6-Diamidin-2-phenylindol
ECs: endothelial cells
ECFCs: endothelial colony forming cells
EdU: 5-ethynyl-2'-deoxyuridine
EGM: Endothelial growth media
EV: extracellular vesicle
FBs: fibroblasts
FBS: fetal bovine serum
FGF-7: fibroblast growth factor 7
FSOs: floating skin organoids
GFP: green fluorescent protein
hiPSC: human induced pluripotent stem cells
hPL: human platelet lysate
hVIM: human vimentin
IL17A: interleukin 17A
K5: keratin 5
K14: keratin 14
KCs: keratinocytes
KGF: keratinocyte growth factor
KGM: keratinocyte growth media
mRNA: messenger ribonucleic acid
PDGF: platelet-derived growth factors
VEGF: vascular endothelial growth factor

## References

[1] Shahbazi MN, Siggia ED, Zernicka-Goetz M (2019). Self-organization of stem cells into embryos: A window on early mammalian development. Science.

[2] Bar-Ephraim YE, Kretzschmar K, Clevers H (2020). Organoids in immunological research. Nat Rev Immunol.

[3] Lancaster MA, Knoblich JA (2014). Organogenesis in a dish: modeling development and disease using organoid technologies. Science.

[4] Yin X, Mead BE, Safaee H, Langer R, Karp JM, Levy O (2016). Engineering Stem Cell Organoids. Cell Stem Cell.

[5] Mansour AA, Gonçalves JT, Bloyd CW (2018). An in vivo model of functional and vascularized human brain organoids. Nat Biotechnol.

[6] Grebenyuk S, Ranga A (2019). Engineering Organoid Vascularization. Front Bioeng Biotechnol.

[7] Takebe T, Wells JM (2019). Organoids by design. Science.

[8] Rheinwald JG, Green H (1975). Serial cultivation of strains of human epidermal keratinocytes: the formation of keratinizing colonies from single cells. Cell.

[9] Takagi R, Ishimaru J, Sugawara A (2016). Bioengineering a 3D integumentary organ system from iPS cells using an in vivo transplantation model. Sci Adv.

[10] Niehues H, Bouwstra JA, El Ghalbzouri A, Brandner JM, Zeeuwen PLJM, van den Bogaard EH (2018). 3D skin models for 3R research: The potential of 3D reconstructed skin models to study skin barrier function. Exp Dermatol.

[11] Lei M, Schumacher LJ, Lai Y-C (2017). Self-organization process in newborn skin organoid formation inspires strategy to restore hair regeneration of adult cells. Proc Natl Acad Sci U S A.

[12] Lee J, Bӧscke R, Tang P-C, Hartman BH, Heller S, Koehler KR (2018). Hair Follicle Development in Mouse Pluripotent Stem Cell-Derived Skin Organoids. Cell Rep.

[13] Lee J, Rabbani CC, Gao H (2020). Hair-bearing human skin generated entirely from pluripotent stem cells. Nature.

[14] Gurtner GC, Werner S, Barrandon Y, Longaker MT (2008). Wound repair and regeneration. Nature.

[15] Ye H, Rahul, Dargar S, Kruger U, De S (2018). Ultrasound elastography reliably identifies altered mechanical properties of burned soft tissues. Burns.

[16] Carulli S, Contin R, De Rosa L, Pellegrini G, De Luca M (2013). The long and winding road that leads to a cure for epidermolysis bullosa. Regen Med.

[17] Jones RE, Foster DS, Longaker MT (2018). Management of Chronic Wounds-2018. JAMA.

[18] De Rosa L, Latella MC, Secone Seconetti A (2020). Toward Combined Cell and Gene Therapy for Genodermatoses. Cold Spring Harb Perspect Biol.

[19] Mavilio F, Pellegrini G, Ferrari S (2006). Correction of junctional epidermolysis bullosa by transplantation of genetically modified epidermal stem cells. Nat Med.

[20] Hirsch T, Rothoeft T, Teig N (2017). Regeneration of the entire human epidermis using transgenic stem cells. Nature.

[21] Andrique L, Recher G, Alessandri K (2019). A model of guided cell self-organization for rapid and spontaneous formation of functional vessels. Sci Adv.

[22] Kaur A, Midha S, Giri S, Mohanty S (2019). Functional skin grafts: Where biomaterials meet stem cells. Stem Cells Int.

[23] Marino D, Luginbühl J, Scola S, Meuli M, Reichmann E (2014). Bioengineering dermo-epidermal skin grafts with blood and lymphatic capillaries. Sci Transl Med.

[24] Chen L, Xing Q, Zhai Q (2017). Pre-vascularization Enhances Therapeutic Effects of Human Mesenchymal Stem Cell Sheets in Full Thickness Skin Wound Repair. Theranostics.

[25] Melero-Martin JM, Khan ZA, Picard A, Wu X, Paruchuri S, Bischoff J (2007). In vivo vasculogenic potential of human blood-derived endothelial progenitor cells. Blood.

[26] Reinisch A, Hofmann NA, Obenauf AC (2009). Humanized large-scale expanded endothelial colony-forming cells function in vitro and in vivo. Blood.

[27] Souidi N, Stolk M, Rudeck J (2017). Stromal Cells Act as Guardians for Endothelial Progenitors by Reducing Their Immunogenicity After Co-Transplantation. Stem Cells.

[28] Gomez-Salinero JM, Rafii S (2018). Endothelial cell adaptation in regeneration. Science.

[29] Italiano JE, Richardson JL, Patel-Hett S (2008). Angiogenesis is regulated by a novel mechanism: pro- and antiangiogenic proteins are organized into separate platelet alpha granules and differentially released. Blood.

[30] Burnouf T, Strunk D, Koh MBC, Schallmoser K (2016). Human platelet lysate: Replacing fetal bovine serum as a gold standard for human cell propagation?. Biomaterials.

[31] Prasain N, Lee MR, Vemula S (2014). Differentiation of human pluripotent stem cells to cells similar to cord-blood endothelial colony-forming cells. Nat Biotechnol.

[32] Muhart M, McFalls S, Kirsner RS (1999). Behavior of tissue-engineered skin: a comparison of a living skin equivalent, autograft, and occlusive dressing in human donor sites. Arch Dermatol.

[33] Laurent J, Blin G, Chatelain F (2017). Convergence of microengineering and cellular self-organization towards functional tissue manufacturing. Nat Biomed Eng.

[34] Griffin MF, DesJardins-Park HE, Mascharak S, Borrelli MR, Longaker MT (2020). Understanding the impact of fibroblast heterogeneity on skin fibrosis. Dis Model Mech.

[35] Herland A, Maoz BM, Das D (2020). Quantitative prediction of human pharmacokinetic responses to drugs via fluidically coupled vascularized organ chips. Nat Biomed Eng.

[36] Brandenberg N, Hoehnel S, Kuttler F (2020). High-throughput automated organoid culture via stem-cell aggregation in microcavity arrays. Nat Biomed Eng.

[37] Scharler C, Poupardin R, Peking P Extra-hematopoietic immunomodulatory role of the SCID-susceptibility gene DOCK-2 identified by stepwise maturation of human iPSCs into clonogenic mesodermal stromal progenitors. 2020; 1-34.

[38] Hochmann S, Mittermeir M, Santic R (2018). Evaluation of modified Interferon alpha mRNA constructs for the treatment of non-melanoma skin cancer. Sci Rep.

[39] Hofmann NA, Reinisch A, Strunk D (2009). Isolation and Large Scale Expansion of Adult Human Endothelial Colony Forming Progenitor Cells. J Vis Exp.

[40] Fusaki N, Ban H, Nishiyama A, Saeki K, Hasegawa M (2009). Efficient induction of transgene-free human pluripotent stem cells using a vector based on Sendai virus, an RNA virus that does not integrate into the host genome. Proc Jpn Acad Ser B Phys Biol Sci.

[41] Kogut I, Roop DR, Bilousova G (2014). Differentiation of human induced pluripotent stem cells into a keratinocyte lineage. Methods Mol Biol.

[42] Olmer R, Engels L, Usman A (2018). Differentiation of Human Pluripotent Stem Cells into Functional Endothelial Cells in Scalable Suspension Culture. Stem cell reports.

[43] Reinisch A, Strunk D (2009). Isolation and animal serum free expansion of human umbilical cord derived mesenchymal stromal cells (MSCs) and endothelial colony forming progenitor cells (ECFCs). J Vis Exp [Internet].

[44] Schallmoser K, Rohde E, Reinisch A (2008). Rapid large-scale expansion of functional mesenchymal stem cells from unmanipulated bone marrow without animal serum. Tissue Eng Part C Methods.

[45] Wang CK, Nelson CF, Brinkman AM, Miller AC, Hoeffler WK (2000). Spontaneous cell sorting of fibroblasts and keratinocytes creates an organotypic human skin equivalent. J Invest Dermatol.

[46] Yang R, Zheng Y, Burrows M (2014). Generation of folliculogenic human epithelial stem cells from induced pluripotent stem cells. Nat Commun.

[47] Reinisch A, Bartmann C, Rohde E (2007). Humanized system to propagate cord blood-derived multipotent mesenchymal stromal cells for clinical application. Regen Med.

[48] Wolf M, Vari B, Blöchl C (2019). Extracellular vesicles from therapeutic grade allogeneic human placental stromal cells induce angiogenesis and modulate immunity. BioRxiv.

[49] Gu Z, Eils R, Schlesner M (2016). Complex heatmaps reveal patterns and correlations in multidimensional genomic data. Bioinformatics.

[50] Peking P, Koller U, Murauer EM (2018). Functional therapies for cutaneous wound repair in epidermolysis bullosa. Adv Drug Deliv Rev.

[51] Schallmoser K, Strunk D (2009). Preparation of Pooled Human Platelet Lysate (pHPL) as an Efficient Supplement for Animal Serum-Free Human Stem Cell Cultures. J Vis Exp.

[52] Ingram DA, Mead LE, Moore DB, Woodard W, Fenoglio A, Yoder MC (2005). Vessel wall-derived endothelial cells rapidly proliferate because they contain a complete hierarchy of endothelial progenitor cells. Blood.

[53] Bartmann C, Rohde E, Schallmoser K (2007). Two steps to functional mesenchymal stromal cells for clinical application. Transfusion.

[54] Shi Y, Inoue H, Wu JC, Yamanaka S (2017). Induced pluripotent stem cell technology: a decade of progress. Nat Rev Drug Discov.

[55] Nielsen MM, Witherden DA, Havran WL (2017). γδ T cells in homeostasis and host defence of epithelial barrier tissues. Nat Rev Immunol.

[56] Correa-Gallegos D, Jiang D, Christ S (2019). Patch repair of deep wounds by mobilized fascia. Nature.

[57] Yoder MC, Mead LE, Prater D (2007). Redefining endothelial progenitor cells via clonal analysis and hematopoietic stem/progenitor cell principals. Blood.

[58] Yang B, Brazile B, Jan N-J, Hua Y, Wei J, Sigal IA (2018). Structured polarized light microscopy for collagen fiber structure and orientation quantification in thick ocular tissues. J Biomed Opt.

[59] Kawamata S, Kanemura H, Sakai N, Takahashi M, Go MJ (2015). Design of a Tumorigenicity Test for Induced Pluripotent Stem Cell (iPSC)-Derived Cell Products. J Clin Med.

[60] Wood FM, Kolybaba ML, Allen P (2006). The use of cultured epithelial autograft in the treatment of major burn wounds: Eleven years of clinical experience. Burns.

[61] Brok-Volchanskaya VS, Bennin DA, Suknuntha K, Klemm LC, Huttenlocher A, Slukvin I (2019). Effective and Rapid Generation of Functional Neutrophils from Induced Pluripotent Stem Cells Using ETV2-Modified mRNA. Stem Cell Reports.

[62] Reinisch A, Etchart N, Thomas D (2015). Epigenetic and in vivo comparison of diverse MSC sources reveals an endochondral signature for human hematopoietic niche formation. Blood.

[63] Reinisch A, Thomas D, Corces MR (2016). A humanized bone marrow ossicle xenotransplantation model enables improved engraftment of healthy and leukemic human hematopoietic cells. Nat Med.

[64] Chan CKF, Gulati GS, Sinha R (2018). Identification of the Human Skeletal Stem Cell. Cell.

[65] Olm F, Lim HC, Schallmoser K, Strunk D, Laurell T, Scheding S (2020). Acoustophoresis Enables the Label-Free Separation of Functionally Different Subsets of Cultured Bone Marrow Stromal Cells. Cytometry A.

[66] Rohban R, Reinisch A, Etchart N (2013). Identification of an effective early signaling signature during neo-vasculogenesis in vivo by ex vivo proteomic profiling. PLoS One.

[67] Hu S, Li Z, Cores J (2019). Needle-Free Injection of Exosomes Derived from Human Dermal Fibroblast Spheroids Ameliorates Skin Photoaging. ACS Nano.

[68] Borena BM, Meyer E, Chiers K (2014). Sphere-forming capacity as an enrichment strategy for epithelial-like stem cells from equine skin. Cell Physiol Biochem.

[69] Woappi Y, Altomare D, Creek KE, Pirisi L (2020). Self-assembling 3D spheroid cultures of human neonatal keratinocytes have enhanced regenerative properties. Stem Cell Res.

[70] Klicks J, Maßlo C, Kluth A, Rudolf R, Hafner M (2019). A novel spheroid-based co-culture model mimics loss of keratinocyte differentiation, melanoma cell invasion, and drug-induced selection of ABCB5-expressing cells. BMC Cancer.

[71] Shyer AE, Rodrigues AR, Schroeder GG, Kassianidou E, Kumar S, Harland RM (2017). Emergent cellular self-organization and mechanosensation initiate follicle pattern in the avian skin. Science.

[72] Kang BM, Kwack MH, Kim MK, Kim JC, Sung YK (2012). Sphere formation increases the ability of cultured human dermal papilla cells to induce hair follicles from mouse epidermal cells in a reconstitution assay. J Invest Dermatol.

