# Supplementary material for: Self-assembly of differentiated progenitor cells facilitates spheroid human skin organoid formation and planar skin regeneration
Source: Theranostics. 2021 Jul 25;11(17):8430–47. doi: 10.7150/thno.59661 (PMC8344006; doi:10.7150/thno.59661)
Supplement: Supplementary file 1 — Supplementary figures and tables. [file thnov11p8430s1.pdf]

Supporting Information:

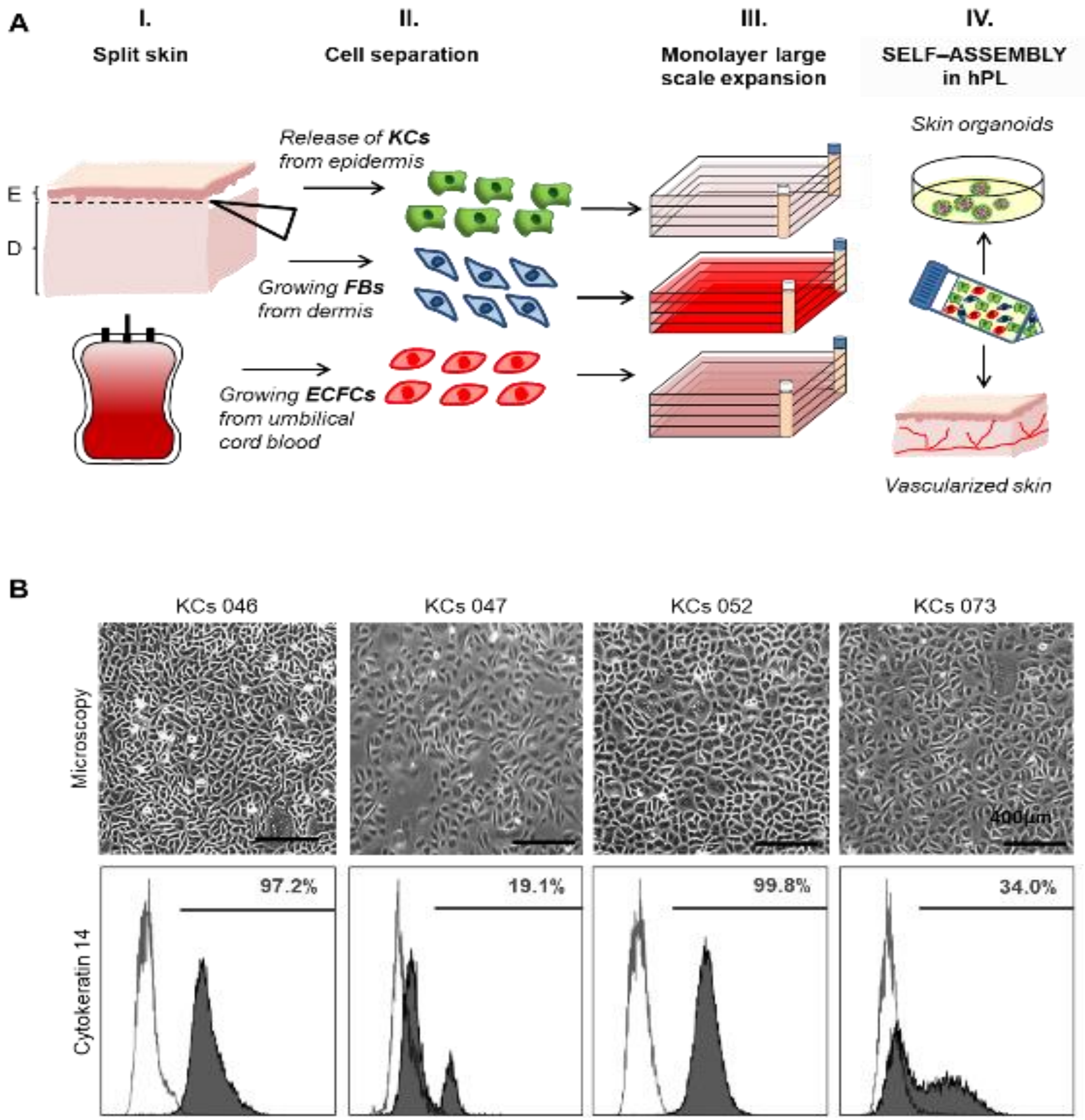

Figure S1: Adult skin cell isolation, characterization and regeneration strategy. (**A**) Illustrative summary of adult keratinocyte (KC), fibroblast (FB) and endothelial colony-forming progenitor cell (ECFC) propagation for testing self-assembly in vitro and in vivo. KCs and FBs were isolated from split-thickness skin explants. ECFCs were isolated from umbilical cord blood (I. + II). All cell types were propagated as monolayers under animal-serum free conditions generating app. 1 x 10^8 cells per cell factory (III.), before generating single-cell suspensions in human platelet lysate (hPL)-supplemented media promoting cell self-assembly into human skin organoids and vascularized human skin (IV.). (**B**) Morphology and keratin 14 expression of four randomly selected KC preparations illustrating donor variability (Scale bar 400 µm). For cell transplantation, >99% pure cytokeratin 14^+ keratinocytes were used.

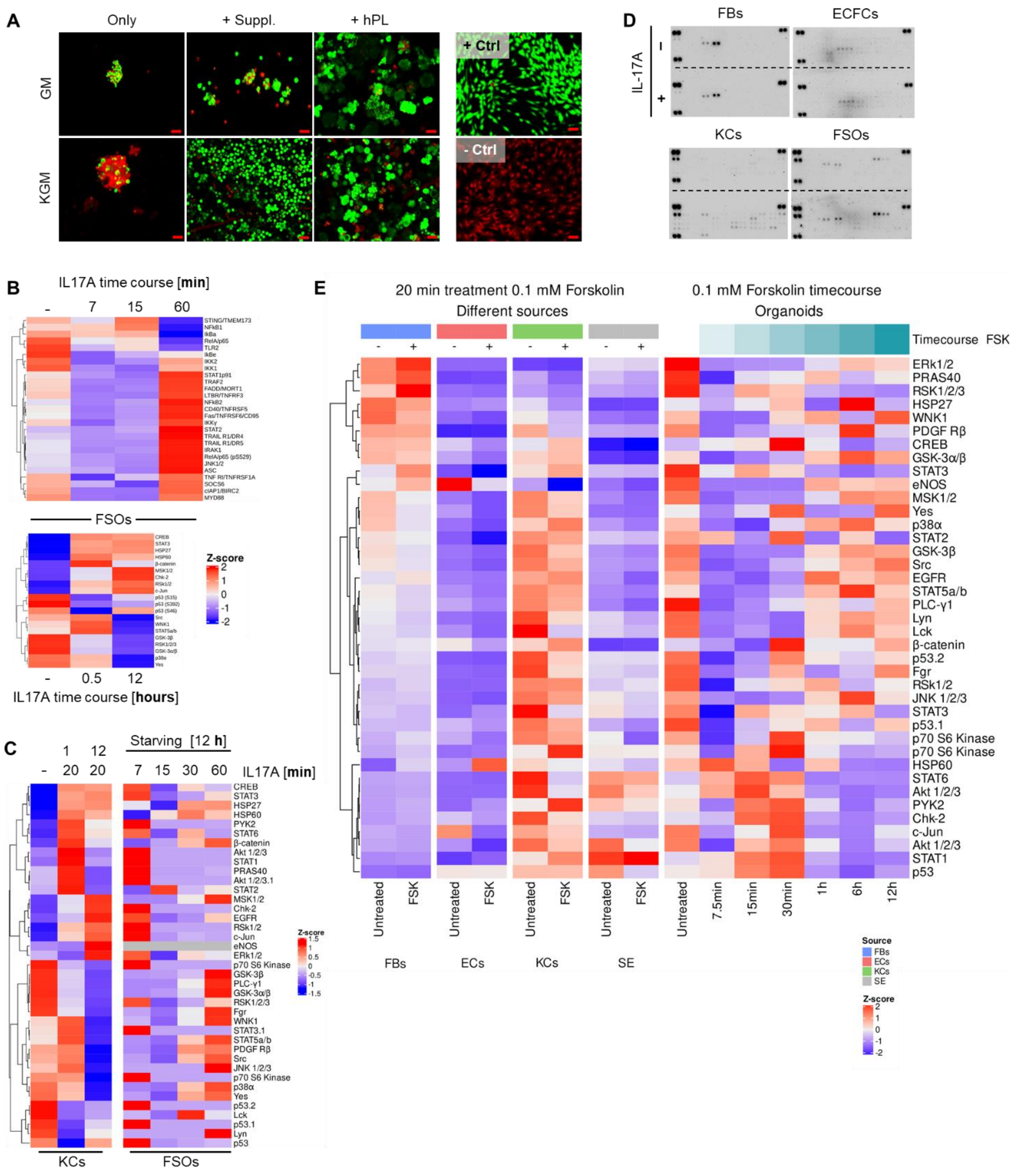

Figure S2: Viability and molecular response of FSOs vs. separated cells to IL-17 and forskolin. (**A**) Life/dead staining (green = viable cells, red = dead cells) showed that viable FSOs are exclusively formed in the presence of human platelet lysate (+hPL; +ctrl, healthy fibroblasts; –ctrl, ethanol treated fibroblasts; scale bar = 50 µm). (**B**) Proteome profiling early (top) and delayed (down) time course heatmap of FSOs in response to IL-17A. (C) Proteome profiling early time course heatmap of starved KCs and FSOs in response to IL-17A. (D) Comparative antibody arrays of single FBs, ECFCs, KCs and FSOs after 12 hour stimulation in the absence (HCl control) or presence of IL17A. (**E**) Proteome profiling of starved single FBs, ECFCs, KCs in response to forskolin (FSK; left) and time course heatmap of 4-day assembled FSOs after 20 min in the absence (-, DMSO solvent control) or presence (+) of FSK (right). Z-score values. (B, C, E) Z-score values.

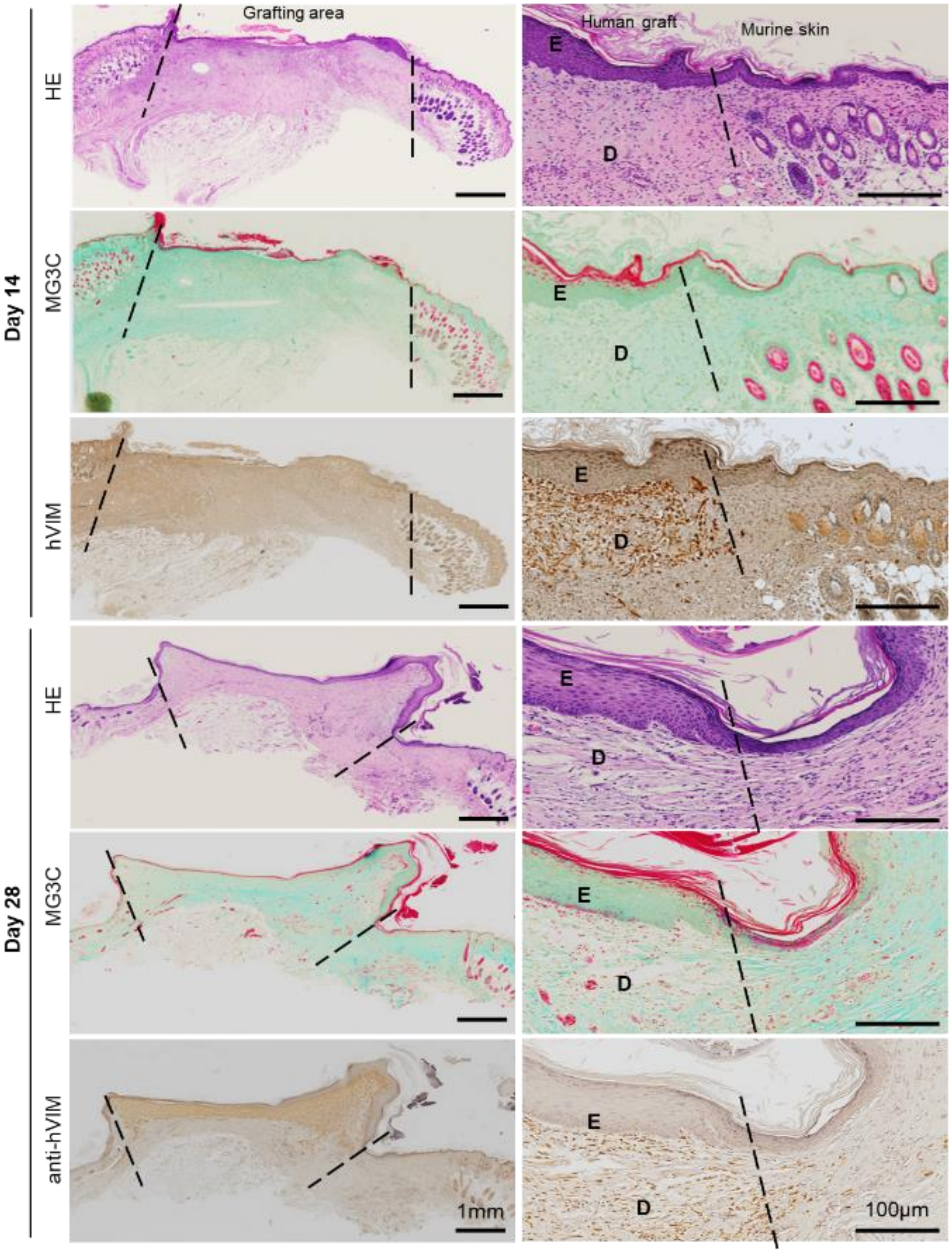

Figure S3: Single-cell suspension transplantation produced layered human skin.

Full scan of skin sections 14 (n = 15 animals) and 28 days (n = 12 animals) after transplantation: Hematoxylin (HE), Masson Goldner trichrome (MG3C) and anti-human vimentin (hVIM) staining showing the grafted area surrounded by murine skin (marked by hatched lines). Representative staining shown.

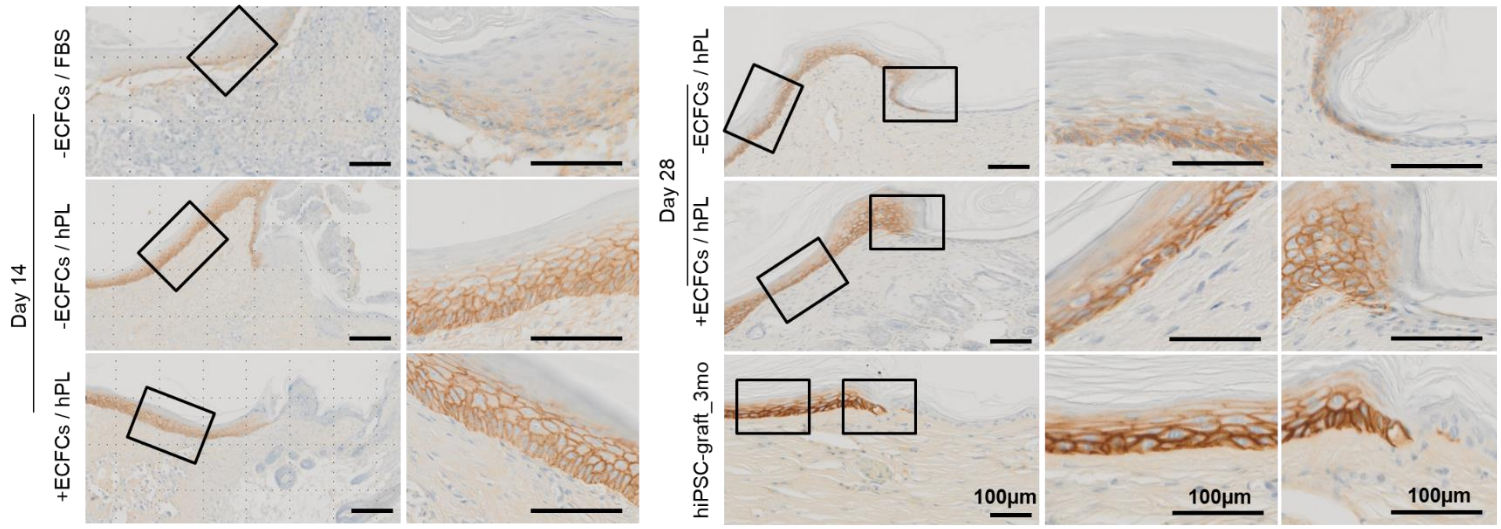

Figure S4: Human origin of epithelium in skin grafts

Anti-human EGFR staining confirmed the human epidermal origin in all transplant groups, showing the border between human (brown staining) and murine epidermis. Higher magnifications of the human epidermis is shown in all transplant groups; higher magnifications of the human/murine borders is shown in 28 day adult cell grafts and hiPSC-graft.

Figure S5 (next page): Histology of transplanted human skin 14 and 28 days after engraftment. (**A**) Layered epidermal-dermal cell organization visualized in hematoxylin/eosin staining (HE). (**B**) Anti-keratin 14 (K14) staining showed keratinocytes located predominantly in the basal epidermal skin layer day 28 after grafting. (**C to D**) Dermal tissue composition in (**C**) HE staining, and (**D**) fiber stain (scale bar, 100 µm; representative staining of one out of three independent grafts per group shown. (**F**) Cell grafts consisting of fibroblasts (FBs) and keratinocytes (KCs) in α-MEM/1% or 10% or 100% human platelet lysate (hPL) were transplanted and analyzed after 14 days. Full section scans of HE stained grafts (overview) showed the human graft surrounded by the murine skin (marked by hatched lines). Higher magnification revealed regular skin cell organization at 10% hPL and bleeding in 100% hPL grafts. Data of one pilot experiment shown (n = 3 animals).

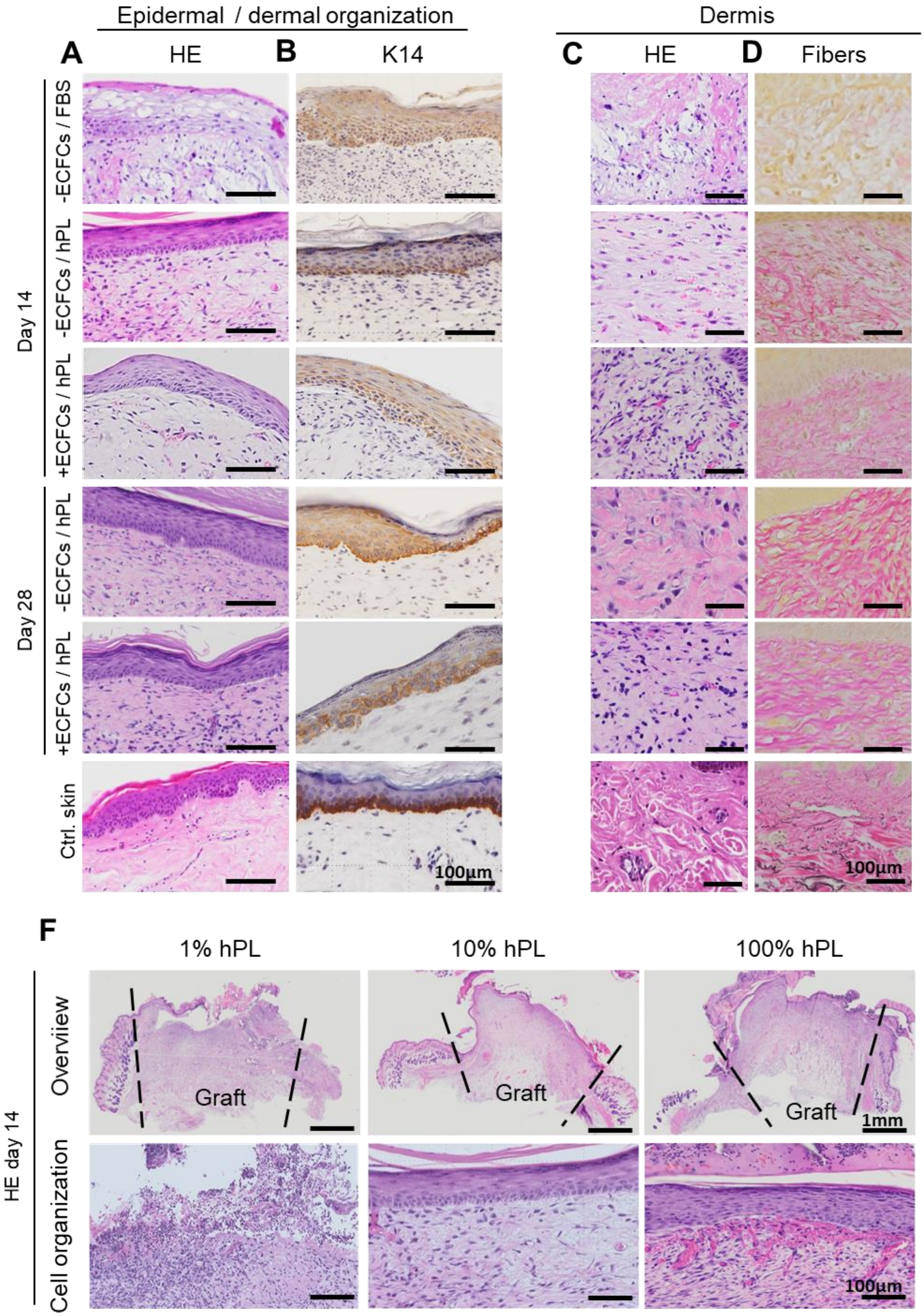

Epidermal / dermal organization
Dermis
A
HE
B
K14
C
HE
D
Fibers
Day 14
-ECFCs / FBS
-ECFCs / hPL
+ECFCs / hPL
Day 28
-ECFCs / hPL
+ECFCs / hPL
Ctrl. skin
100µm
100µm
F
1% hPL
10% hPL
100% hPL
HE day 14
Overviiew
Cell organization
Graft
Graft
Graft
1mm
100µm

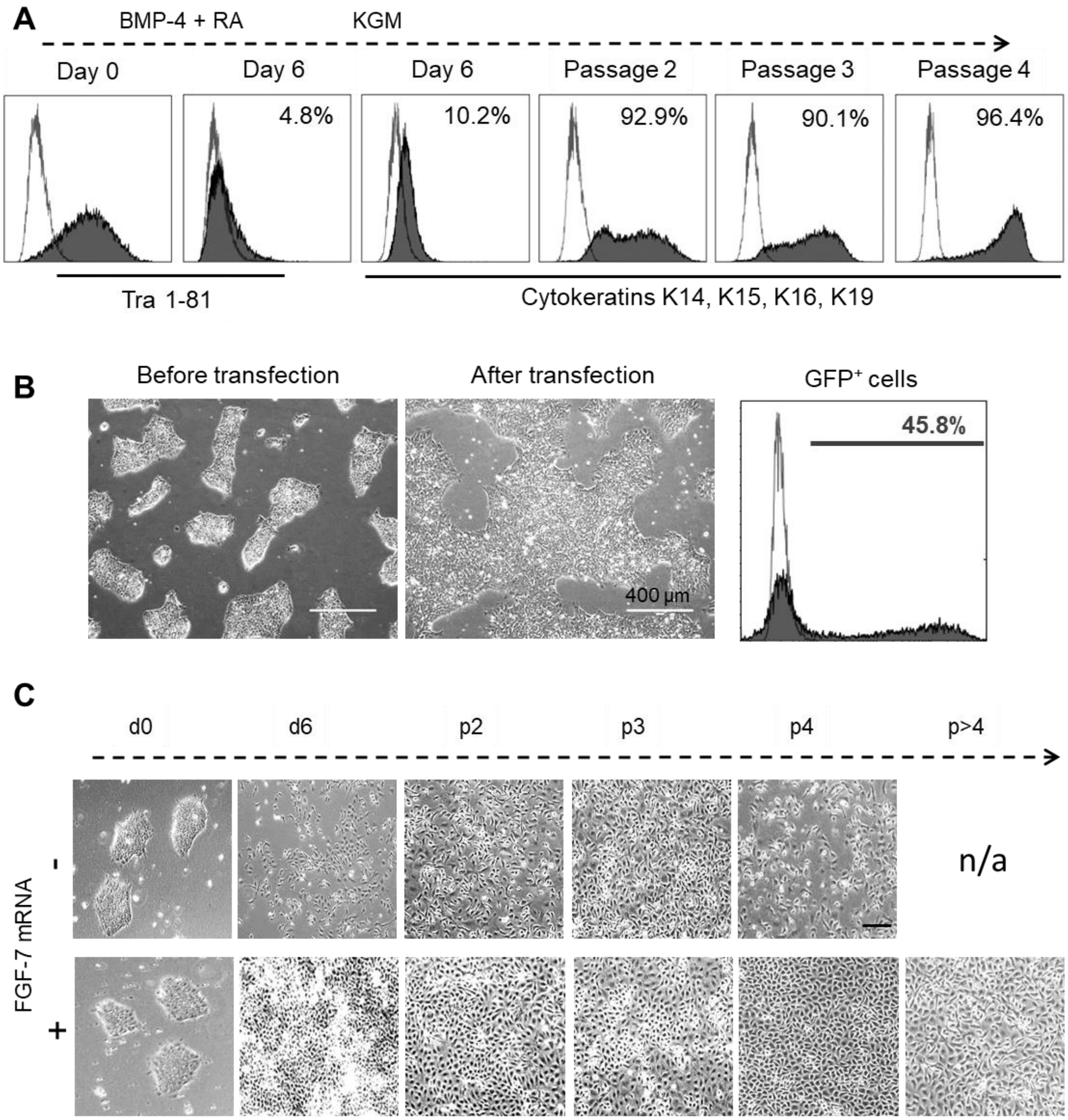

Figure S6: Keratinocyte differentiation from hiPCSs without FGF-7. (**A**) Reproducing the keratinocyte differentiation protocol containing BMP-4 and RA during the differentiation phase [41]. Flow cytometry revealed a reduction of pluripotency marker Tra-1-81 until day 6 of differentiation and a stepwise increase in keratinocyte marker expression during maturation from day 6 to passage 4. (**B**) Stabilized mRNA transfection of hiPSCs: Transient transfection of hiPSC clones using a GFP mRNA showed normal cell growth one day after transfection, resulting in 45.8% efficiency 24 hours after transfection. (**A and B**) Data of one pilot experiment shown (n=1). (**C**) KGF mRNA-transfected hiPSC-KCs showed an extended passaging potential (p > 4) compared to hiPSC-KCs created according to the standard protocol. Representative images shown (n=2 independent hiPSC clones).

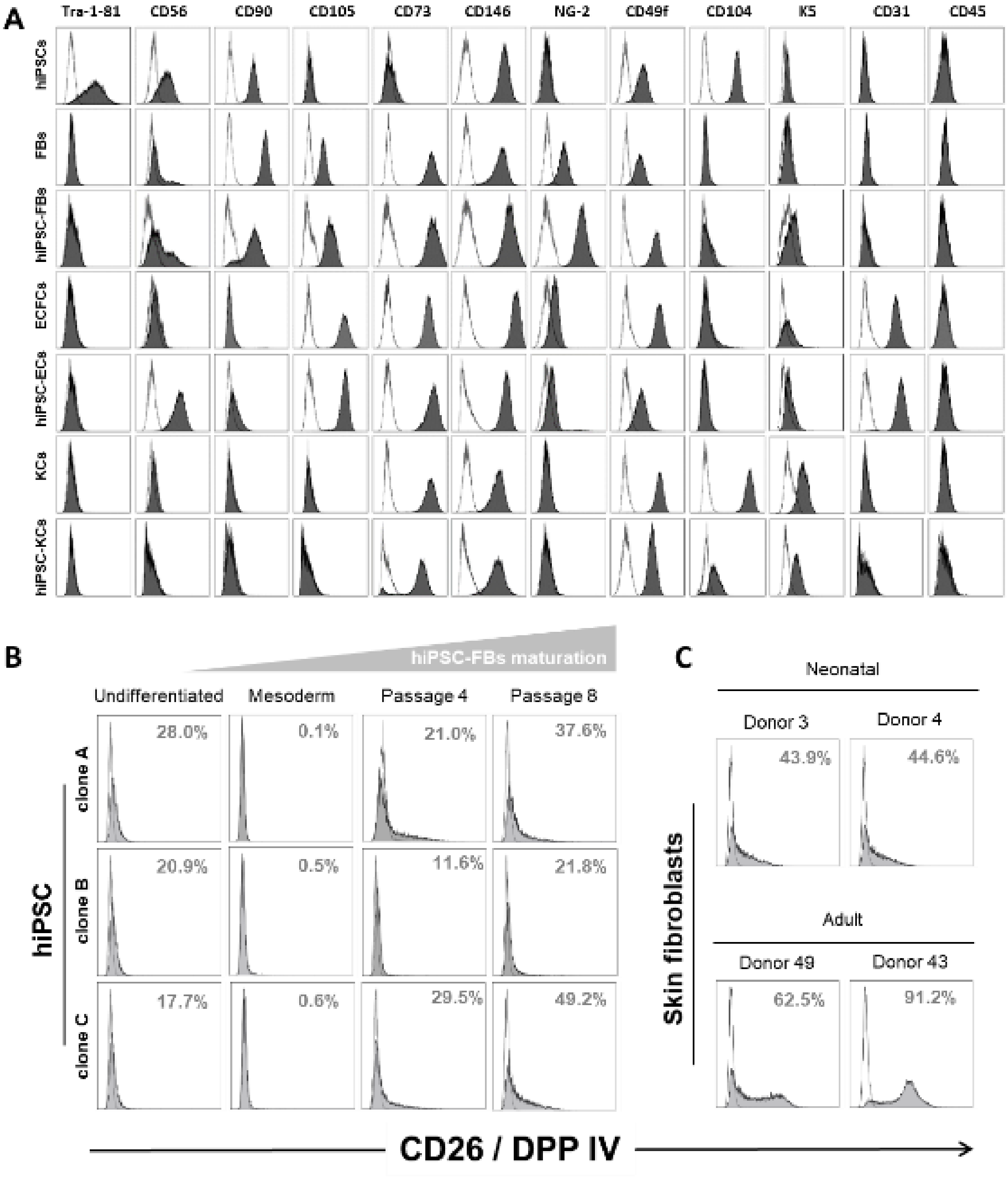

Figure S7: Phenotypic analysis of hiPSC-derived and adult cells and monitoring of hiPSC-derived, neonatal and adult fibroblast's CD26 expression. (**A**) Flow cytometry showed comparable phenotypes of hiPSC-derived cells and their corresponding adult cell types (n = 1 to 3). (**B**) Increase of CD26 expression during hiPSC-FB maturation, confirming a consecutive transition from a hypothetically regenerative ($CD26^-$) to scarring ($CD26^+$) fibroblast phenotype during cell maturation. (**C**) CD26 expression levels of neonatal and adult fibroblasts compared to hiPSC-FBs. Two out of three donors shown.

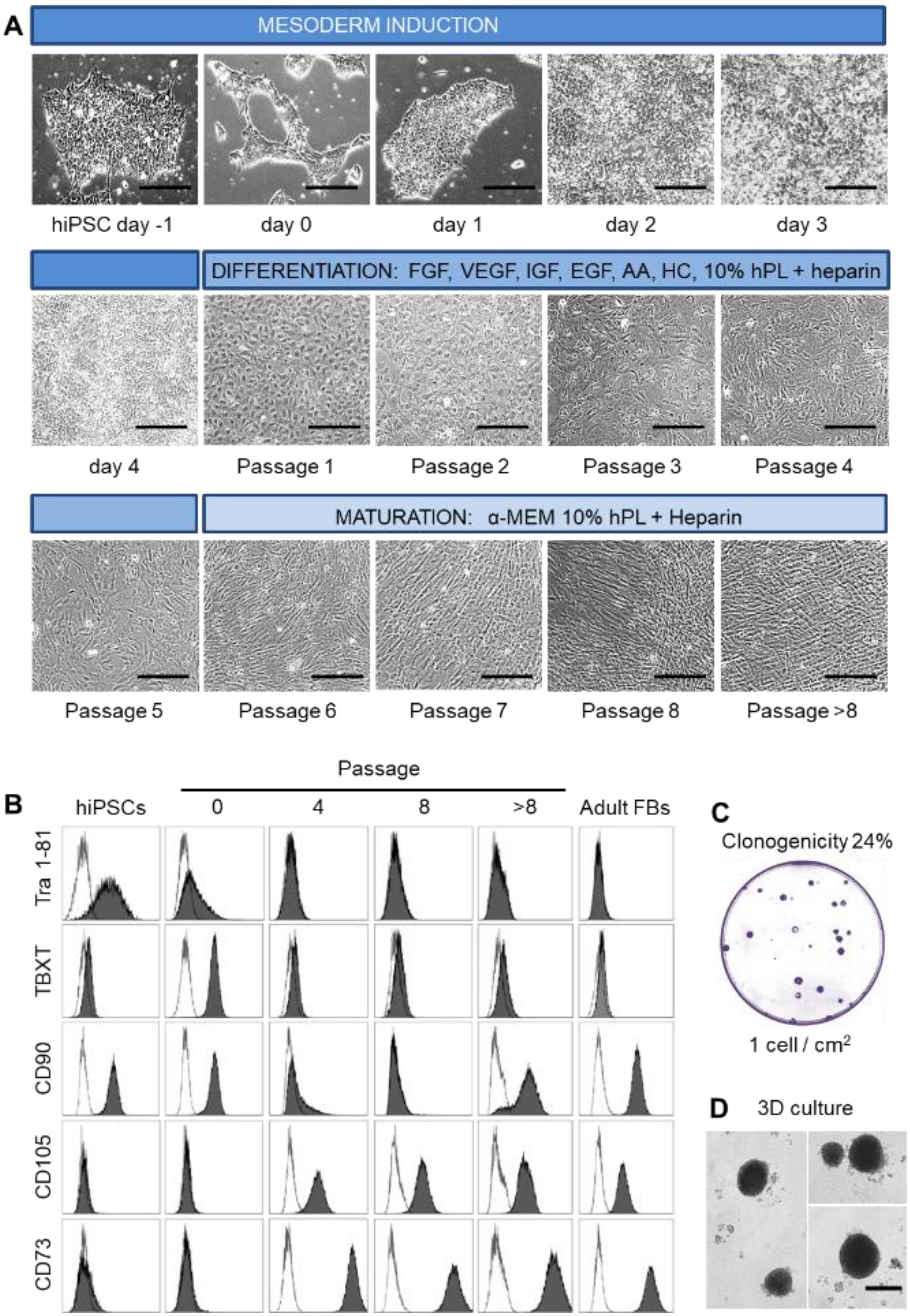

Figure S8: Mesoderm induction, differentiation and maturation of fibroblasts from hiPSCs. (**A**) Morphological changes of hiPSCs during four days of mesoderm induction, followed by five passages of early fibroblast (FB)

differentiation and 4 - 6 passages of FB maturation. **(B)** Phenotypic analysis showed the stepwise differentiation and maturation of hiPSC-FBs differentiation, resulting in Tra 1-81$^-$, TBXT/brachyury$^-$, CD90$^+$, CD73$^+$ and CD105$^+$ mature hiPSC-FBs assuming a phenotype otherwise comparable to adult FBs. (**C**) Colony-forming unit (CFU) assay showed clonogenic potential of hiPSC-FBs. (**D**) 3D culture of hiPSC-FBs (representative pictures shown). (**A - D**) Representative data from two independent hiPSC clones (n=2).

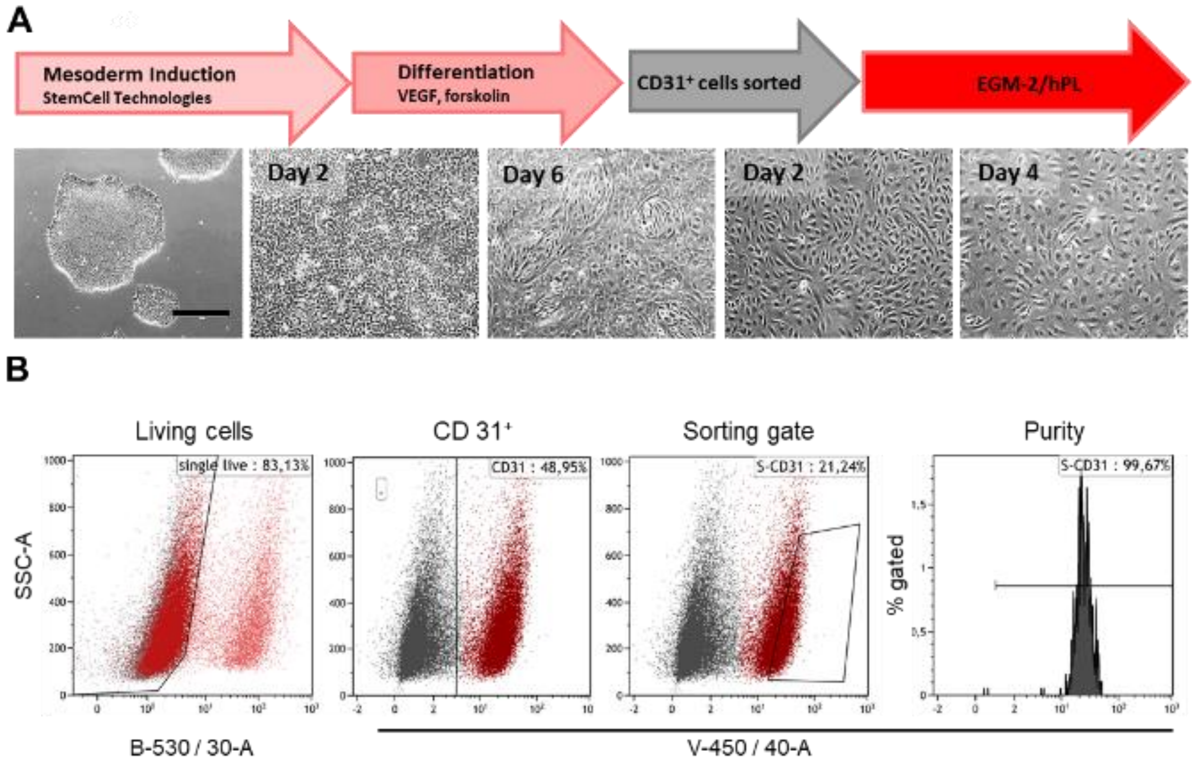

Figure S9: Mesoderm induction, differentiation and maturation of endothelial cells from hiPSCs. (**A**) Morphological changes of hiPSCs during two days of mesoderm induction, followed by four days of endothelial differentiation. Day six CD31$^+$ cells were purified by fluorescence-activated cell sorting before continuation of endothelial cell maturation**.** (**B**) Sorting strategy: Viable single cells in this randomly selected sample contained 48.9% CD31$^+$ cells in a bulk population at day 6 during differentiation. The sorting gate included 22.8% CD31$^+$ cells, resulting a sort purity of 99.5%. (**A and B**) Representative data from one out of three independent hiPSC clones shown (n=3).

Table S1: Parameters for evaluating the dermal score.

**A) Vascularization and hemorrhage**	
No blood vessels / no hemorrhage	0
Strong hemorrhage (with or without blood vessels)	1
Blood vessels / low to moderate hemorrhage	2
Blood vessels / no hemorrhage	3
**B) Cell density / ground substance produced**	
High cell density	0
Moderate cell density / no ground substance	1
Moderate cell density / moderate ground substance	2
Low cell density / distinct ground substance	3
**C) Thickness and density of fibers**	
No fibers	0
Thin or thick fiber / high fiber density	1
Thin fiber / moderate to low fiber density	2
Thick fiber / moderate to low fiber density	3

Table S2: Summary of transplant groups and key parameters tested.

Absolut numbers or mean ± SD given.

**Transplant group**	**Cells**	FBs+KCs	FBs+KCs	FBs+KCs+E CFCs	FBs+KCs	FBs+KCs+E CFCs	Human skin
	**Medium**	10% FBS	10% hPL	10% hPL	10% hPL	10% hPL	-
	**Time point**	14 days	14 days	14 days	28 days	28 days	adult
**Transplant quality**	**Human origin**	4/4	3/4	4/4	3/3	3/4	-
	**Cell organization**	3/4	3/4	4/4	3/3	3/4	-
	**Epidermal thickness [µm]**	203.3 ± 45.2	150.0 ± 49.7	128.0 ± 21.3	138.6 ± 35.7	112.2 ± 32.0	113.3 ± 11.9
	**Collagen fibers [n]**	1/4	3/4	4/4	3/3	3/4	3/3
	**Ground substance**	1/4	3/4	4/4	3/3	3/4	3/3
	**Vessels / 0.1 mm²**	1.5 ± 1.3	3.7 ± 2.3	6.8 ± 3.3	6.8 ± 2.3	5.8 ± 2.6	1.5 ± 1.2
	**Dermal score**	2.0 ± 1.2	5.0 ± 1.5	6.2 ± 1.7	7.6 ± 1.5	6.6 ± 1.3	8.6 ± 0.9

Table S3: **Parameters addressed to evaluate tumorigenic potential of hiPSC-derived cells** (according to published work [59]).

	Parameter	Description	Outlined
**Cells**	Cell production	Cell culture research lab and expansion in CF4	Fig. 4, 5, Fig. S8
	Quality control of cells	Cell type specific phenotype, lack of pluripotency markers	Fig. S7a
	Cell dose of transplanted cells	$3x10^6$ KC, $3x10^6$ FB, $3x10^6$ ECFCs	Fig. 6, Material & Methods
**Application**	Type of immunodeficient animal model	NOD.Cg-Prkdc^{scid} Il2rg^{tm1Wjl}/SzJ B and T cell deficient; dysfunctional NK cells	https://www.jax.org/strain/005557
	Method of transplantation	Single cell suspension transplant in grafting chamber onto muscle fascia of mice, subcutaneously	Fig. 6
	Gender and numbers of animals	six female mice	Material & Methods
	Monitoring periods	2 weeks and 12 weeks post transplantation	Material & Methods
**Tumorigenesis**	Histology	Alu probing to detect human cell nuclei in the transplant	Fig. 6
		H&E and Ki67 showed no tumor formation	Fig. 6
	Analysis sites	Skin	Fig. 6, Material & Methods
	Positive control	hiPSCs were injected into NSG mice and induced teratoma	Scharler *et al.*

Table S4. List of antibodies for flow cytometry.

Name	Host	Clonality	Clone	Working concentration	Isotype	Company	Catalog no
Tra 1-81-AF647	mouse	monoclonal	TRA-1-81	60 ng/ml	IgM, k	BD Biosciences	560793
SSEA-4-PE	mouse	monoclonal	MC813-70	60 ng/ml	IgG3	BD Biosciences	560128
OCT4-PE	mouse	monoclonal	3A2A20	2.5 µg/ml	IgG2b, k	biolegend	653704
Nanog-PE	mouse	monoclonal	N31-355	78 ng/ml	IgG1, k	BD Biosciences	560483
CD56-PE	mouse	monoclonal	CMSSB	1.25 µg/ml	IgG1, k	eBioscience	12-0567
CD90-BUV395	mouse	monoclonal	5E10	4µg/ml	IgG1, k	BD Biosciences	563804
CD105-eFluor450	mouse	monoclonal	SN6	1 µg/ml	IgG1, k	eBioscience	48-1057
CD73-PE	mouse	monoclonal	AD2	376 ng/ml	IgG1, k	BD Biosciences	550257
CD146-PE/ Vio770	mouse	monoclonal	541-10B2	1.1 µg/ml	IgG1	Miltenyi	130-099-956
NG2-APC	mouse	monoclonal	LHM-2	2 µg/ml	IgG1	R&D Systems	FAB2585A
CD49f-BV421	rat	monoclonal	GoH3	1.25 ng/ml	IgG2k (rat)	BD Biosciences	562582
CD104-PE	rat	monoclonal	439-9B	1.25 ng/ml	IgG2b	BD Biosciences	555720
CD45-APC	mouse	monoclonal	HI30	120 ng/ml	IgG1, k	BD Biosciences	555485
CD31-eFluor450	mouse	monoclonal	WM59	500 ng/ml	IgG1, k	eBiosciences	48-0319-42
Cytokeratin 14, 15, 16, 19 AF647	mouse	monoclonal	KA4	2 µg/ml	IgG1	BD Biosciences	563648
Brachyury-APC	goat	polyclonal		200 ng/ml	IgG	R&D Systems	IC2085A
CD26-APC	mouse	monoclonal	M-A261	1 µg/ml	IgG1, k	BD Biosciences	560223
P63 unlabeled	mouse	monoclonal	4E5	10 µg/ml	IgG1	abcam	ab110038

Cytokeratin 14 unlabeled	mouse	monoclonal	LL001	4 µg/ml	IgG2a	Santa Cruz	sc-53253
Cytokeratin 5 unlabelled	mouse	monoclonal	2C2	20 µg/ml	IgG1	Thermo Fischer	MA5-17057
anti-mouse Ig-FITC	goat	polyclonal	-	50 µg/ml	lg	BD Biosciences	554001
anti-mouse Ig-PE	goat	polyclonal	-	40 µg/mL	Ig	BD Biosciences	550589
